# Supplementation with Lentil (*Lens culinaris*) Hull Soluble Dietary Fiber Ameliorates Sodium Dextran Sulfate-Induced Colitis and Behavioral Deficits via the Gut-Brain Axis

**DOI:** 10.3390/foods14050870

**Published:** 2025-03-03

**Authors:** Dongying Chen, Xin Bi, Qian Feng, Yong Sun

**Affiliations:** 1 The Second Affiliated Hospital, Jiangxi Medical College, Nanchang University, Nanchang 330006, China; 407900220056@email.ncu.edu.cn; 2 State Key Laboratory of Food Science and Resources, Nanchang University, Nanchang 330047, China; bixin@email.ncu.edu.cn

**Keywords:** lentil hulls, soluble dietary fiber, colitis, cognitive impairment, sphingolipid metabolism, gut-brain axis

## Abstract

In this study, the impact of lentil hull soluble dietary fibers (SDFs) on colitis and behavioral deficits in mice was assessed. Structural characterizations of SDFs confirmed that cellulase-modified soluble dietary fiber exhibited better physicochemical properties: more porous microstructure; similar polysaccharide structure; more stable particle size distribution; higher crystallinity; better adsorption capacity; and lower viscosity. Additionally, we explored its potential cognitive benefits via the gut-brain axis by behavioral tests, histopathology, 16S rRNA sequencing, gas chromatography and metabolomics analysis. The results showed that SDFs significantly improved inflammatory symptoms in colon and brain and cognitive behaviors. LSDF had better efficacy than HSDF. LSDF intervention decreased the harmful bacteria abundance (*Bacteroides*, *Flexispira* and *Escherichia*, etc.) and increased beneficial bacteria abundance (*Aggregatibacter* and *Helicobacter*, etc.). LSDF also affected brain metabolites through the sphingolipid metabolism. Spearman correlation analysis showed that there was a positive correlation between harmful bacteria with inflammatory factors (LPS, IL-1β, IL-6, and TNF-α, etc.) and sphingolipid metabolites, while beneficial bacteria were positively correlated with brain-derived neurotrophic factor (BDNF), IL-10, and cognitive behavior. This study highlights the value of SDFs in future diet-based therapeutic strategies targeting gut-brain interactions.

## 1. Introduction

Pulses have long been of great interest in developing functional foods for health all over the world, and lentils (*Lens culinaris* L.) are a globally crucial traditional dietary legume, with about a quarter of their yield coming from Canada [1]. The dehulling process of lentils generates 20–28% of the total lentil processed hulls as a low-value by-product, resulting in the wastage of a promising health-promoting food ingredient—dietary fiber [2]. Lentil hulls are composed of 60–90% dietary fiber, 3% ash, 2–8% protein, and 1–3% lipids, making them a rich source of prebiotics [2]. The basic components of dietary fiber include pectin, cellulose, mannans, and xylans [3].

Dietary fibers from different sources have distinct chemical structures and fermentation properties, with significant variability in their ability to modulate inflammation in inflammation bowel disease (IBD) [4]. IBD is a group of chronic inflammatory bowel diseases (IBDs) that primarily include ulcerative colitis (UC) and Crohn’s disease (CD). This study focused on the effect of lentil bean hull SDFs on mice UC and its cognitive impairment through gut-brain axis. Based on its solubility in hot water, dietary fiber can be further classified into two types: insoluble dietary fiber (IDF) and soluble dietary fiber (SDF), each with unique biological functions. SDF consists of a number of oligosaccharides and indigestible polysaccharides such as pectin, inulin, Arabic gum, and β-glucan, while lignin, hemicellulose, and cellulose constitute IDF [5]. Compared to IDF, SDF provides greater viscosity during food processing and is commonly used as an emulsifier or gelling agent [6]. As a unique polysaccharide, SDF has been shown to enhance the abundance of Bifidobacterium in the gut, effectively alleviating colitis [7]. SDF acts as a fermentation substrate for microorganisms, producing short-chain fatty acids (SCFAs), which have been proven to reduce inflammation and reinforce the intestinal mucosal barrier, thereby alleviating UC inflammation [8,9]. Meanwhile, SDF intake has also been found to improve cognitive performance. For instance, 5% fructans were found to be effective in altering the gut microbiota structure and increase SCFA levels, enhancing cognitive function in the male Alzheimer mice [10]. However, research on how SDF from natural plant sources, such as lentil hulls, affects brain function and behavioral aspects in the alleviation of colitis, and how brain metabolite reciprocally affect colitis, remains limited.

Ulcerative colitis (UC) has become a global health burden, with an estimated 5 million cases worldwide in 2023 [11]. According to epidemiological research, the incidence rates are stabilizing or decreasing in the majority of nations with high incomes in North America, Northern Europe, and Australia, while sharply increasing in countries with low or middle incomes in Asia [11]. Treatment options for UC include aminosalicylates for mild to moderate cases, immunosuppressants for moderate to severe cases, and steroids for acute episodes [12]. The current therapeutic goals for UC focus on halting disease progression and preventing inflammation-induced flares [13]. However, these medications often have side effects and limited efficacy, driving interest in plant-sourced dietary treatment with fewer adverse effects. Many studies have identified that SDF could assist those diagnosed with colitis. SDF from Quinoa bran was proven to alleviate the symptoms of colitis and improve the diversity of gut microbial community [14]. SDF has been reported to exhibit higher antioxidant capability, thermal stability, and water-holding capacity than cellulose. These characteristics might be explained by the altered monosaccharide content, thinner particle size, and more porous structure of SDF. SDF was subsequently found to reduce the clinical signs of colitis mice based on these functional characteristics. [15].

Gut microbiota not only affects the host’s immune system [13], but also contributes to the synthesis of metabolites and neuroactive factors [16]. Studies have shown that certain members of the *Bacteroidetes* are able to break down polysaccharides with higher molecular weights, and then those small molecular weight sugars are utilized by the Firmicutes to provide energy for other microorganisms [17]. *Akkermansia muciniphila* has been shown to hydrolyze carbohydrates from the colon’s mucus layer, using them as substrates to generate acetate and oligosaccharides [18,19]. SCFAs also have important effects on the nervous system. For instance, studies have demonstrated that sodium butyrate promotes the expression of BDNF, neural proliferation, and neurogenesis of mice [20,21]. Propionate is important for normal brain development and behavioral control, which is implicated in neurodevelopmental disorders such as autism [22,23].

The primary mediators of gut–brain axis communication are metabolites (e.g., SCFAs), the vagus nerves, hormones (e.g., hypothalamic–pituitary–adrenaline), and immune activation which are neurological pathways generated by the gut microbiota [24]. The disruption of the gut microbiota structure alters the concentrations of inflammatory agents and metabolites, including lipopolysaccharides (LPSs) and pro-inflammatory cytokines, which then cause altered levels of neuroinflammation by increasing the permeation of the gut and the barrier between brain and blood [25]. Emerging evidence demonstrates that peripheral inflammatory factors in IBD patients also promote the development of various disorders of the central nervous system, including depression and anxiety [26]. The opening of the gut vascular barrier, which connects the gut and the liver, can spread gut inflammation to the brain, which can result in mental impairments like anxiety and depression [27]. An essential scientific technique for researching the gut–brain axis mechanism is metabolomics. A study assessed the relationship of brain neurotransmitters and gut metabolites in gnotobiotic mice in vivo based on an LC-MS/MS-based targeted metabolomics approach [28]. Recently, there has been a growing interest in investigating the gut–brain axis in relation to cognitive and colitis issues. For instance, through the microbial-gut-brain axis, the carotenoid component lycopene (LYC) has been found to have a beneficial preventive effect on colitis and associated disorders of behavior [29]. Apple polyphenols have been demonstrated to repair colitis and related cognitive deficits via strengthening the gut barrier, reducing inflammation, repairing neurological impairment and gut microbiota composition, and modulating circadian rhythms [30]. Furthermore, probiotic supplementation suppressed neuroinflammation in DSS-induced colitis mice by reducing inflammation, as well as modulating neurotransmitters [31].

Current research to develop interventions and therapeutics targeting related sphingolipids is generating interest. Glycerophoslipids, polyunsaturated fatty acids, and sphingolipids are among the substances that are crucial in causing depressive symptoms [32]. Moreover, sphingolipids, lysophospholipids, and phosphatidylcholine were among the metabolites that were found to be altered in the brains of sad mice in earlier research [33]. Sphingomyelin (SM), ceramide (CER), sphingosine (SPH), and sphingosine-1P (S1P) are important metabolites of some sphingolipid metabolic pathways. Recent metabolomics suggests that sphingomyelin (SM) contributes as a biomarker for IBD diagnosis [34]. Ceramides (CERs) are a component of the intestinal epithelial cell membrane and may serve as a prospective biomarker for UC [35]. Levels of specific ceramide subgroups (e.g., C18:0-Cer, C20:0-Cer, and LacC16-Cer) are closely related with UC, this might be helpful in differentiating UC patients from healthy individuals [36]. Sphingosine is involved in signaling processes related to cell survival and neurodegeneration, with sphingomyelin and ceramide being the precursors of sphingosine, which is phosphorylated by SPHK gene to produce S1P [37]. According to recent research, sphingosine-1P (S1P) has a role in the pathophysiology of disorders affecting the brain, particularly those involving the transmission of synaptic information, neuroinflammation, and neuronal autophagy [38]. Overall, one potential therapeutic approach for the treatment of depression has been the modulation of the sphingolipid metabolism.

Although some studies have explored how dietary fiber affects intestinal health and cognitive decline, studies investigating the mechanism of action of SDFs extracted from plant sources on colitis through the gut–brain axis are still very limited [4,8,10]; hence, this study aims to fill this gap. This is the first study to characterize the structure of SDFs from lentil hulls, in particular, comparing the structural differences between SDFs obtained by two different enzymatic methods, which reveals the structural basis of their mechanism in alleviating colitis and cognitive impairment. We then utilized the sodium dextran sulfate (DSS)-induced cognitive impairment in a mouse model of colitis to assess the functional activity of SDFs. Using microbiomics and a metabolomic analysis of brain tissues, we explored the potential mechanisms by which SDFs in lentil hulls regulate metabolic pathways and inflammatory responses through the gut–brain axis. This study is the first to investigate the nutritional effects of SDFs derived from lentil hulls on DSS-induced colitis and behavioral deficits in mice, and provides a better theoretical basis for future population-based experiments.

## 2. Materials and Methods

### 2.1. Materials and Reagents

The Canadian International Grains Research Institute (Winnipeg, MB, Canada) supplied green lentil hulls. Lentil hulls were dried at 60 °C, crushed with a small high-speed pulverizer (Hebei Benchen Science and Technology Co., Ltd., Hebei, China), the raw material for this study were then acquired after passing through 120-mesh filter. The materials were kept at −4 °C after being finely powdered. MP Biomedicals (Irvine, CA, USA) provided the dextran sodium sulfate (36–50 kDa). Aladdin (Shanghai, China) provided reference stand of SCFAs. We purchased BDNF and LPS ELISA kits from Jiangsu Meimian Industrial Co., Ltd. (Yancheng, China).

SDFs include two types of soluble dietary fiber: SDF and SDFM. In this study, SDF is a soluble dietary fiber obtained using classical enzymatic methods [39], specifically using three enzymes, including thermostable α-amylase, protease, and saccharolytic enzyme. On the other hand, SDFM is modified soluble dietary fiber obtained with the addition of cellulase, specifically using four enzymes, including thermostable α-amylase, protease, saccharolytic enzyme, and cellulase. The full description of the SDF and SDFM preparation process: initially, 10 g dried lentil hulls were ground and sieved (200 μm), the milled hulls were sequentially mixed with petroleum ether (1:25 *w*/*v*) and 85% ethanol (1:10 *w*/*v*) with homogenizer to remove the fat and free sugar, respectively. After drying in a vacuum oven, the hulls were stirred and suspended in phosphate buffer salts (PH = 6, 1:20 *w*/*v*) and then the mixture was ultrasonically modified in an ultrasonic machine (35 min, 15 °C, 100 W). Then, the samples were hydrolyzed with the addition of 2.5 mL thermostable α-amylase (PH = 6, 5 min, 100 °C). After cooling to 50 °C, 30 mg protease (PH = 6) was added and the mixture was hydrolyzed for 35 min. The PH was adjusted to about 4.5 with the addition of 5 mL 3 mol/L acetic acid and then 30 μL of saccharolytic enzyme was added, followed by hydrolyzing for 30 min at 60 °C. The solution was boiled for 10 min to deactivate the enzymes and then centrifuged at 4000× *g* for 15 min. The supernatant was decanted and filtered through a sintered glass funnel containing diatomaceous earth, then evaporated to 1/4 of the original volume using a rotary evaporator at 65 °C. The concentrated supernatant was mixed with 4 times the supernatant volume of 95% ethanol (preheated to 60 °C), and SDF was precipitated by refrigeration at 4 °C overnight and centrifuged at 4500× *g* for 10 min. Thereafter, the residue was redissolved in water and subjected to the spinning step once more and freeze-dried. The dried samples were washed with 100 mL of 95% ethanol and acetone, and subsequently dried in a vacuum oven at 80 °C, there after ground to powder and stored in a desiccator. SDFM was synthesized by adding 30 mg of cellulase and incubating at 50 °C for 1.5 h following the completion of hydrolysis by the saccharase enzyme. Both the preceding and following steps were consistent with those previously described.

### 2.2. Structural Characterizations and Functional Properties of SDFs

#### 2.2.1. Scanning Electron Microscopy (SEM)

SDFs were examined at 5 kV for surface and microstructure using an SEM (Quanta200F, FEI, Hillsboro, OR, USA). Prior to observation, the dehydrated samples were covered with a 100 μm thick coating of gold after being sprinkled on a support using double-sided conducting adhesive tapes. SDF micrographs were captured at a magnification of 3000×.

#### 2.2.2. Fourier Transfer-Infrared Spectrometry (FT-IR)

FTIR analysis of SDFs was obtained by a Thermo Nicolet 5700 instrument (Thermo Fisher Scientific, Waltham, MA, USA). The SDF powder was blended with potassium bromide powder (1:100, *w*/*w*), and the scan range was set from 400 to 4000 cm^−1^ during 32 scans.

#### 2.2.3. Particle Size

The distribution of particle sizes of SDFs was measured using a laser particle size analyzer (Malvern Mastersize 2000, Malvern Panalytical Ltd., Malvern, UK). SDFs was formed into a solution of 1 mg/mL concentration with ultrapure water, and then, the results were tested at room temperature after 20 min of sonication.

#### 2.2.4. X-Ray Diffraction (XRD)

To identify the crystalline state of SDFs, an X-ray diffractometer (Empyrean, Malvern Panalytical, Almelo, The Netherlands) was employed. With a resolution of 0.02° and a scan angle of 2θ = 5–60°, it was run at 10 mA and 30 kV. To determine the relative crystallinity of SDFs, the Segal method was applied [40].

#### 2.2.5. Adsorption Capacity of Glucose and Cholesterol

Some studies reported that SDF may be able to modulate intestinal microbial metabolism by adsorbing glucose to the concentrations of metabolites including SCFAs, which in turn improves the intestinal inflammatory response [41,42]. Moreover, it has been found that cognitive impairment is closely related to hyperglycemia [43], and by regulating blood glucose levels, SDFs may also play an indirect role in the protection of cognitive function. Referring to the method of [44], 0.5 g SDF was melted in 50 mL of glucose solution (100 mM/L) and then incubated at a thermostatic shaking incubator (37 °C, 6 h), centrifuged (4500 rpm/min, 15 min), the concentration of glucose of supernatant was detected at 505 nm using the DNS colorimetric method. The glucose concentration in the initial glucose solution was recorded as *a* and the supernatant’s glucose concentration (*b*) was noted. The following formula was used to calculate the glucose adsorption capacity (*GAC*) (unit: mg/g):

(1) $$GAC=\frac{a-b}{m}$$

The study reported that intestinal concentrations of cholesterol are linked to intestinal inflammation [45], and cognitive impairment is also thought to be closely linked to abnormalities in the metabolism of lipids [46]. SDFs were added into two egg yolk solution (fresh egg yolk mixed with 9× volume distilled water), whose pH was adjusted to 2 and 7, respectively, shaken (80 rpm/min, 37 °C, 2 h) with a shaker, centrifuged (15 min, 4500 rpm/min), and left, after which the phthalaldehyde approach was used to determine the supernatant’s cholesterol level. Using the following formula, the cholesterol adsorption capacity (*CAC*) (unit: mg/g) was determined, where *C_1_* and *C_0_* are the cholesterol content of the egg yolk solution and its supernatant after centrifugation without SDFs, respectively; *C* is the cholesterol content of the supernatant after the addition of SDFs.

(2) $$CAC=\frac{[\left(C_{0}-C\right)-\left(C_{1}-C_{0}\right)]\times 25}{m}$$

#### 2.2.6. Flow Behavior

##### Static Rheology of SDFs

At 55 °C, the SDFs were dissolved in distilled water to a 40 mg/mL concentration while being continuously stirred for 30 min. To equilibrate and release any trapped air, the solution was then kept at 4 °C for 12 h. A DHR-2 rheometer (TA Instruments, New Castle, DE, USA) is employed to determine the solution’s viscosity at shear rates between 0.1 and 100/s followed by equilibration at 25 °C for 5 min.

##### Dynamic Rheology of SDFs

Test samples were prepared by dissolving 100 mg SDFs in 10 mL distilled water with constant stirring. The SDF gel was then poured into a rheometer plate and allowed to equilibrate for 2 min. Silicone oil droplets were placed at the border of cone to prevent evaporation throughout tests. To measure changes of stored energy modulus elasticity (G′) and loss modulus viscosity (G″) with angular frequency from 0.1 to 10 Hz, strain scanning tests were conducted using a DHR-2 rheometer (TA Instruments, New Castle, DE, USA) at 25 °C and 1% strain at a range of 0.1–10 Hz.

### 2.3. Animals, DSS-Induced Colitis Model, and SDF Intervention

**Figure 1:**
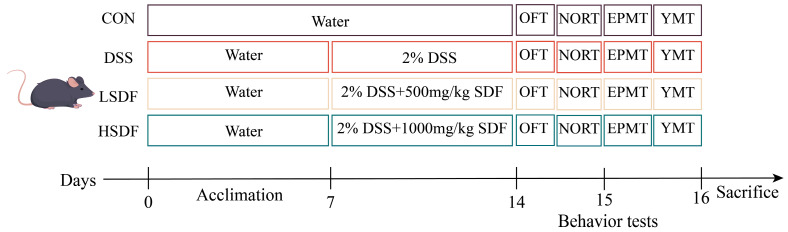
The protocol scheme diagram of colitis mouse model.

We acquired 48 male C57BL/6J mice from SPF Bio-Technology Co., Ltd. that were 6–8 weeks old. Mice lived under conditions of a 12 h light/dark cycle, a temperature of 20 ± 2 °C, and a humidity of 55 ± 5%. Each cage contained 4 mice. The Ethics Committee approved the use of animals in the Jiangxi University of Chinese Medicine Experimental Animal Science and Technology Centre (JZLLSC20220492). After 7 days of acclimation and housing under controlled conditions, to examine the effect of SDFs on UC, each of the four groups (*n* = 12 mice) was randomly assigned to either CON, DSS, LSDF treatment (Gavage 500 mg/kg/d of b.w. SDF), or HSDF treatment (Gavage 1000 mg/kg/d of b.w. SDF). All mice were given the standard diet (AIN-93G), the CON group mice were given distilled water for the duration of the trial, whereas the mice in the remaining groups received distilled water for seven days prior to receiving 2% DSS for seven days. On the 15th day, the open field test (OFT) and new object recognition test (NORT) were administered. The elevated plus maze test (EPMT) and Y-maze test (YMT) was conducted on the 16th day. Figure 1 displays the animal experimental design.

#### 2.3.1. Behavioral Tests

##### OFT

**Figure 2:**
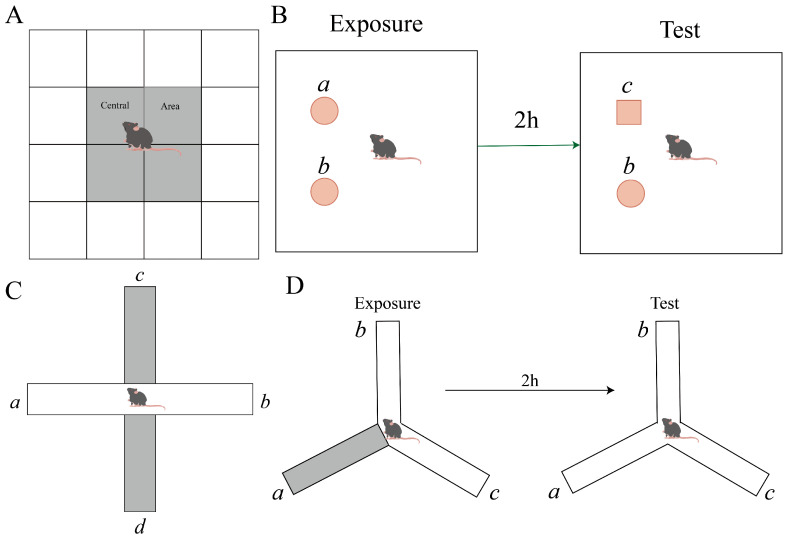
Diagrammatic representation of experimental setup: open field test (**A**), new object recognition test (**B**), elevated plus maze test (**C**), and Y-maze test (**D**).

The OFT was conducted to estimate the locomotor activity and anxious behavior in mice. After being first positioned in a corner, each mouse was afforded 10 min for unrestrained exploration. The box was virtually divided into sixteen quadrans, with the four central sections specifically marked as the central sections. The specific device schematic is shown in Figure 2A. KEMaze software version number 1.0 (Nanjing Karvin Biotechnology Co., Ltd., Nanjing, Jiangsu, China) was used to record and examine the overall distance travelled, the number of admissions into the center, and the duration of time spent there.

##### NORT

NORT is based on animals’ innate interest in novelty and uses their exploratory behavior towards new objects to measure their ability to perceive and remember changes in their environment. At the beginning, the mice were put in a square box containing two similar cylinders *a* and *b*. Two hours later, the mice were reintroduced to the same environment; however, a new square item, *c*, took the place of one of the original objects, *a*. The mice were free to explore the environment the entire time and interact with the two similar objects. Usually, more time spent exploring the new object is seen as an indicator of better memory function. The specific device schematic is shown in Figure 2B.

##### EPMT

Two opposed open arms (*a* and *b*, each measuring 6 cm × 30 cm) and two opposed closed arms (*c* and *d*, each measuring 6 cm × 10 cm × 30 cm) constituted the EPMT device. The specific device schematic is shown in Figure 2C. The device was positioned 70 cm above the floor. Every mouse was put in the middle of the device (6 cm × 6 cm), facing with the open arm, and each test lasted for 5 min. The number of entries, total dwell time, and distance in open arms (*a* and *b*) were recorded within 5 min.

##### YMT

The mice’s spatial cognitive performance was assessed using the Y-maze test. Three identical arms, each measuring 28 cm in length, 10 cm in height, and 5 cm in width, were joined at a 120° angle. A specific device schematic is shown in Figure 2D. During the learning period, the *a* arms was closed, each mouse was set at the terminus of the *b* arm and given three minutes to wander around. Arm *a* was open two hours later, and then the earlier process was carried out once more. Reduced anxiety-like behaviors were indicated by more entrances and more time spent in open arms.

#### 2.3.2. Disease Activity Index (DAI) Assessment

In this investigation, the DAI score was calculated by averaging the three indicators: rectal bleeding, stool consistency, and weight loss [39], and the details are shown in the Table S1.

#### 2.3.3. Experimental Records and Sample Collection

During the experimental, the mice’s body weight was recorded every day. Blood was obtained by orbital blood sampling and centrifuged to obtain the supernatant serum. Then, the mice underwent cervical dislocation after being anesthetized with isoflurane on the sixteenth day. After that, the brain, feces, colon, and colon contents were all systematically gathered. Colonic length was obtained by measuring with a stainless steel straightedge.

#### 2.3.4. Serum LPS and BDNF Level Detection

The ELISA kits (Wuhan Elabscience Co., Ltd., Wuhan, China) were utilized to measure the concentrations of BDNF and lipopolysaccharide (LPS) in serum.

#### 2.3.5. Hematoxylin and Eosin (H&E) Staining of Colon and Brain

To evaluate the histological damage of colitis and brain, the whole brain and a small fragment of the colon (0.5 cm) were collected. Using the appropriate procedures, sections embedded in 5 μm paraffin were made and analyzed with H&E staining. Images of the staining results were obtained by a camera (Nikon Co., Tokyo, Japan).

#### 2.3.6. Real-Time Quantitative Polymerase Chain Reaction (RT-qPCR)

By applying the RNAeasy^TM^ Animal RNA Isolation Kit (Beyotime Biotech Inc., Shanghai, China), total RNA was isolated from the mice’s brain and colon tissue according to the manufacturer’s guidelines. Following the use of a Nano Drop 8000 quantifier (Thermo Fisher Scientific) to verify the RNA’s quantity and purity, all the RNA was diluted to the same concentration. The PrimeScript RT Reverse Transcription Kit (Takara Biotechnology Co., Shiga, Japan) was used to reverse-transcribe RNA to generate cDNA, using a PCR Thermal Cycler (T100RT, Bio-Rad Laboratories, Inc., Hercules, CA, USA), with the following cycle parameters: 25 °C for 5 min, 50 °C for 15 min, 85 °C for 5 min, and ultimately 4 °C for 10 min. Using a Fluorescent Quantitative PCR Instrument (CFX Connect^TM^), RT-qPCR was carried out, and the primer sequence list for BDNF, TNF-α, Tlr4, IL-6, NF-κB, IL-10, IL-1β, SGMS1, SGMS2, CERS4, CERS6, SPHK1, and SPHK2 was provided on Table S2.

#### 2.3.7. Gut Microbiota Analysis

Gene sequencing was commissioned to Personal Biotechnology Co., Ltd. (Shanghai, China). First, DNA from the gut microbiota was obtained and tested for concentration and purity, according to the manufacturer’s instructions. The V3–V4 region of the 16S rRNA gene was subsequently amplified using PCR. The amplicon paired end 2 × 250 bp sequencing was purified, quantified, and then sequenced. The output results were then examined on the QIIME2 platform.

#### 2.3.8. Measurements of Fecal SCFAs

According to the previous method [39], 100 mg of the fecal sample or the stand solution was combined with 700 μL deionized water, followed by adding 35 μL of 10% sulfuric acid solution and sonicating for 10 min in an ice bath (100 W). After being centrifuged for 15 min (10,000× *g* and 4 °C), the supernatant was obtained for analysis. A 0.22 μm filter membrane was used to filter the combined supernatant after the precipitate was mixed with 0.7 mL of water and the previous step was repeated. The content of SCFAs were detected using a gas chromatograph and the DB-WAX capillary chromatographic column (30 m × 0.25 mm × 0.25 μm, Lanzhou, China). The supernatant was injected into the inlet at 240 °C with an 8:1 split ratio. The temperature methodology was identical to the manufacturer’s instructions, as described in a previously published reference [47]. Using the standard curve, sample peak areas were determined to represent the SCFA content in feces.

#### 2.3.9. Untargeted Metabolomics Analysis

In short, twenty milligrams of cerebral cortical brain tissues were homogenized with 400 μL ice-cold methanol on a TissueLyzer (Qiagen, Hilden, Germany) at 60 Hz for 3 min and then followed by an hour of incubation at −20 °C. After centrifugation for 10 min (15,000× *g* and 4 °C), the supernatant was carefully aggregated, blow-dried, and then resuspended in 50 μL ACN–water = 2:98. The primary metabolites profiled in the brain were identified by the Q Exactive Focus system equipped with a Thermo-Accucore C18 column (2.1 × 100 mm; 2.6 μm, Waters, Waltham, MA, USA). Acetonitrile (B) and formic acid (A) at 0.1% made up the mobile phase. Table S3 displays the specific gradient elution settings. The mobile phase flow rate was 0.4 mL/min, the injection volume was 2 μL, and the column temperature was 40 °C. The following parameters were set for the ESI source: capillary temperature at 325 °C, spray voltage at 2.75 kV, aux gas heater temperature at 400 °C, and scanning mass range at 80–1000 m/z, a full MS resolution at 70,000, and sheath gas flow rate at 60 mL/min, aux gas flow rate at 10 mL/min, and sweep gas flow rate at 1 mL/min.

Progenesis QI (version 2.4) was used to transform the raw MS data, and measurements were made of peak detection, matching, and comparison. Initially, the Human Metabolome Database (HMDB) was applied in order to annotate the metabolites. Orthogonal partial least-squares discriminant analysis (OPLS-DA) and pareto-scaled principal component analysis (PCA) were conducted with Simca (version 14.1). The variables of importance (VIP) were calculated to assess each variable’s contribution to categorization in the OPLS-DA model. The significance of the differences between groups was evaluated using the Student’s *t*-test. To determine whether metabolites may be classified as differential metabolites, the following criteria were used: VIP value > 1, *p*-value < 0.05, and |Fold Change| > 2. Biomarker screening and functional annotation analysis (KEGG pathway annotation analysis, metabolic pathway enrichment analysis) were subsequently employed to investigate the significantly different metabolites. Spearman correlation analysis was utilized to evaluate the relationship between intestinal microbiota and markers linked to colitis and cognition.

### 2.4. Statistical Analysis

All results were expressed as the mean ± standard error of the mean (X ± SEM). SPSS 26.0 (SPSS Inc., Chicago, IL, USA) was used for the statistical analysis of the experimental data. One-way analysis of variance was used for statistical significance evaluated, followed by Duncan test to evaluate the variations in data between groups. For 16S rRNA sequencing and metabolomics data, a Wilcoxon rank sum test and *t*-test were used for significant differences among different groups, respectively. When the *p*-value < 0.05, the data were identified as statistically significant difference.

## 3. Results

### 3.1. Scanning Electron Microscopy Observation

**Figure 3:**
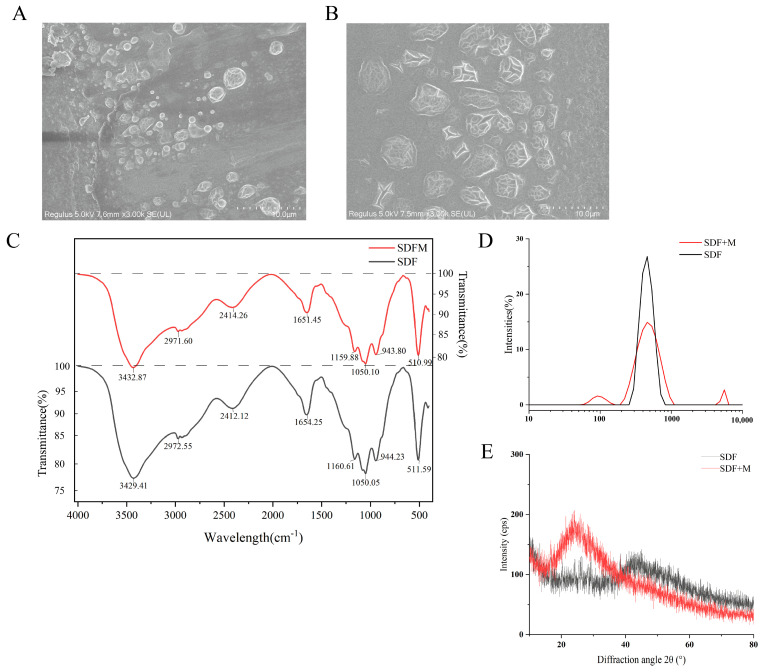
The structural characterizations and functional properties of SDFs. (**A**) The scanning electron microscopy of SDF, scale bar: 10 μm; (**B**) The scanning electron microscopy of SDFM, scale bar: 10 μm; (**C**) The Fourier transfer infrared spectrometry of SDF and SDFM; (**D**) The particle size of SDF and SDFM; and (**E**) The X-ray diffraction spectrometry of SDF and SDFM.

In this study, soluble dietary fiber was extracted by two methods, mainly to investigate the effect of cellulase addition on the structural properties of soluble dietary fiber from lentil hull. SDFM (modified soluble dietary fiber) was extracted with cellulase addition, while SDF (soluble dietary fiber) was extracted with classic enzymatic methods without cellulase. Figure 3A,B show the different properties of SDF and SDFM under 3000× magnification. It can be observed that the particles of SDF and SDFM were round, oval in shape and small. SDFM showed the enzymatic hydrolysis of the cellulose effect with more cracks appearing on surface and in a larger size compared to SDF. The changes in the microstructure of SDFM compared to SDF were probably a result of the enzymatic process of cellulase. The enzymatic digestion may cleave the SDFM surface into more crevices and lead to the aggregation of SDFM extracted from lentil hulls. Surface morphology results may be associated with diverse structures. A more sparse and porous structure means that the SDFM has increased the specific surface area, which enables it to more effectively adsorb substances such as cholesterol and glucose [48].

### 3.2. Fourier Transform Infrared Spectroscopy (FT-IR) of SDFs

Figure 3C’s infrared absorption spectra demonstrate that the wavelength distributions of soluble dietary fiber (SDF) and modified soluble dietary fiber (SDFM) are similar. The wide absorption signals of SDF and SDFM at around 890 cm^−1^ indicate that glucose has a β configuration [49]. Each of these peaks was a typical cellulose structure peak. For both samples, the main peaks at about 3400 cm^−1^ and 2500 cm^−1^ are attributed to the C–H absorption of the rings and the O–H stretching absorption carried on by the intramolecular and intermolecular hydrogen bonding of uronic acids, respectively [50]. And, the absorption peak near 3400 cm^−1^ usually corresponds to the stretching vibration of C–H. The presence of C–H helps soluble dietary fiber to form a gel-like substance in the intestinal tract [51], delaying the absorption of sugars and lipids, and thus showing the biological activities of lowering blood glucose and blood lipids. The spectrum area in the 900–1200 cm^−1^ range, which represents the skeletal C–O and C–C vibration bands of glycosidic bonds and the pyranose rings, reflects similarities in the neutral sugar content of the separated polysaccharides [52]. At the same time, the absorption peaks between 900 and 1200 cm^−1^ correspond to the stretching vibration of C–O, indicating that soluble dietary fiber contains hemicellulose, which contributes to the structural stability and adsorption capacity of dietary fiber [53]. Furthermore, there are bands of absorption at 2972 cm^−1^ (asymmetric bending) and 2862 cm^−1^ (symmetric bending) which correspond to the C–H of the methyl ethers of galacturonic acid. The prominent absorption band of soluble dietary fiber at 1050 cm^−1^ were attributed to C–C, C–O, and the glycosidic C–O–C stretching, which are commonly reported for xylans and arabinoxylans [54,55]. The presence of these functional groups indicates that SDFs have a characteristic absorption peak with a polysaccharide structure, which contributes to the water-holding and swelling properties of the dietary fiber [56], thus enhancing its bioactivity in regulating intestinal function and lowering cholesterol.

### 3.3. Particle Size Analysis Results of SDFs

The results of the particle size of soluble dietary fiber from lentil hulls are shown in Figure 3D. The average particle size of the two samples was around 500 nm, and the particle size distribution map of SDFM showed one large single peak and two small peaks, while SDF showed only one large single peak, which exhibited a more uniform particle size distribution system. The appearance of two small peaks indicates that there is a new oligosaccharide or monosaccharide production in the cellulose enzymatic digestion process, which changes the molecular structure of SDFM and results in a higher surface area and more active sites [57]. In addition, the newly produced oligosaccharides or monosaccharides can act as prebiotics to promote the growth and reproduction of beneficial intestinal bacteria. And, they can produce short-chain fatty acids after being fermented in the intestines [58], thus regulating the balance of intestinal microbiota and enhancing intestinal health.

### 3.4. X-Ray Diffraction (XRD) Analysis Results of SDFs

Figure 3E showed the X-ray diffraction (XRD) intensity profiles and crystallinity variation of soluble dietary fiber (SDF) and modified soluble dietary fiber (SDFM) samples. According to XRD analysis, SDF exhibited a strong diffraction peak at approximately 45° and weak diffraction peak at approximately 24°, while SDFM only had a strong diffraction peak at 24°. A faint diffraction peak that emerged at around 42.66° belonged to a characteristic diffraction peak of cellulose type I [59]. The typical cellulose I type diffraction angles, with a strong peak in the (002) diffraction direction at 24°, might have overlapped with the hemicellulose peak [59,60]. The diffraction peak intensities of the SDFM samples were generally higher than those of the SDF samples, suggesting that the addition of cellulase increased the diffraction intensities of some specific crystal planes. And the cellulase-treated sample’s peak was sharper at 24° than the untreated sample, indicating that the enzymatic hydrolysis of cellulase improved crystallinity, similar to the previous reports [61]. The core of crystalline cellulose is made up of a linear chain of β (1–4) connected D-glucopyranose units. There are two portions of it: crystalline and amorphous [62]. Hemicellulose is typically attached to cellulose microfibrils, but has a random and amorphous structure with low strength and is easily hydrolyzed by several hemicellulases [63]. The increase in crystallinity is primarily attributed to the hydrolysis of hemicellulose and the amorphous portion of cellulose [61,64]. The degree of crystallinity of SDFs is an important structural characteristic that influences their biological activity [65]. Higher crystallinity results in higher stability and a longer residence time in the gut [66], thus enhancing its ability to adsorb substances such as cholesterol and glucose. In addition, it has been shown that changes in crystallinity affect the antioxidant capacity of soluble dietary fiber. For example, the soluble dietary fiber of coffee peer-modified by ultrasound had increased crystallinity and its free radical scavenging activity was significantly enhanced [67]. These property changes may have implications for the treatment of colitis and cognitive impairment.

### 3.5. Glucose and Cholesterol Adsorption Capacity

**Figure 4:**
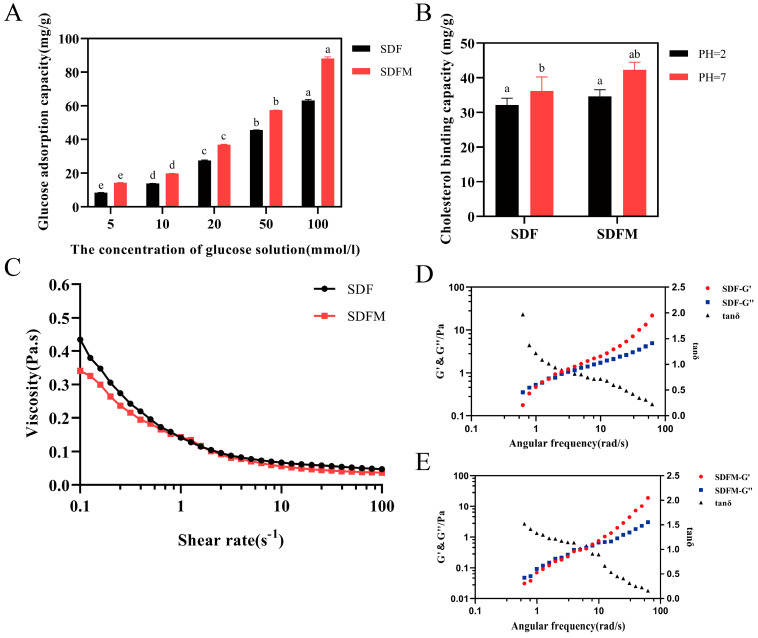
The structural characterizations and functional properties of SDFs. The glucose (**A**) and cholesterol (**B**) adsorption capacity of SDF and SDFM; (**C**–**E**) The flow behavior characteristic of SDF and SDFM, including static rheology of SDFs (**C**) and the dynamic rheology of SDF (**D**) and SDFM (**E**). The data are displayed as mean ± SEM. Statical analyses were carried out using one-way ANOVA along with Duncan’s multiple range test. Different letters indicate significant differences (*p* < 0.05).

Research has shown that there is a strong link between blood sugar levels and cognitive function [68]. SDFM had a greater adsorption ability on glucose than SDF, and this difference became more noticeable with the increase in concentration (Figure 4A). By increasing the glucose adsorption ability, SDFM reduces the intestinal absorption of glucose, which may help control the blood glucose levels and maintain stable blood glucose levels, which are necessary for cognitive function. There are numerous studies which have shown that dietary fiber lowers blood cholesterol [69]. Different mechanisms underlie soluble dietary fiber’s ability to decrease cholesterol. The binding of water in the food and the resulting increase in viscosity are assumed to be the major effect [70]. Some other studies have demonstrated that there is a directly binding force between SDF and cholesterol, which reduces the risk of cardiovascular disease [71]. From our findings, soluble dietary fiber significantly increased the cholesterol absorption capacity at pH 7 compared to pH 2, which corresponded to oral digestive, upper small intestinal, oral digestive, and gastric digestive conditions, respectively. Furthermore, the cholesterol absorption capacity of SDFM was significantly higher than that of SDF (Figure 4B). SDFs adsorb cholesterol from the intestines and reduce its absorption into the bloodstream, thus helping to lower blood cholesterol levels. This may be beneficial in preventing cardiovascular disease, which studies have shown to be associated with cognitive impairment [72], which may explain why SDFs can alleviate cognitive impairment.

### 3.6. The Rheological Characteristic

#### 3.6.1. The Steady State Rheology of SDFs

The rheological properties of SDF and SDFM are characterized under stress, which induce changes in flow behavior and structure. Figure 4C shows the steady-state rheological properties of SDF and SDFM at 40 mg/mL. The results show that SDFM has a lower viscosity, which is due to the difference in monosaccharide composition. Based on the previous results of monosaccharide composition, it can be seen that the structure of each monosaccharide composition of SDFM changed after cellulase treatment, and the viscous sugars have a significant effect on the static rheology of soluble dietary fiber. Within a shear rate of 0–100 s^−1^, the SDF and SDFM viscosities vary inversely to the change in shear rate, which showed the characteristics of a pseudoplastic fluid. When the shear rate increases, the molecules of SDF and SDFM tend to be orientated in a directional arrangement, the interaction between molecules is weakened, the solution mobility increases and the viscosity decreases. It can be expected from the trend in the figure that, after more than 100 s^−1^, the SDF solution will exhibit Newtonian fluid behavior, which suggests that the viscosity does not change with the shear rate, probably because the high shear rearranges the molecular skeleton of SDF and SDFM, making it difficult for the small particles of the SDF and the SDFM to be entangled with each other, which is reflected in the reduction in viscosity [73].

#### 3.6.2. The Dynamic Rheology of SDFs

The dynamic viscoelastic properties of SDF and SDFM samples at 10 mg/mL concentration at angular frequencies from 0.1 to 100 rad/s are shown in Figure 4D,E. The elastic modulus G′ indicates the ability of the material to undergo elasticity, while the viscous modulus G” indicates the viscous characteristics of the material [74]. Both G′ and G″ of SDF and SDFM increase with the angular frequency, and when the angular frequency of SDF < 2, G′ always < G″, which indicates that SDF behaves predominantly viscous in solution. This phenomenon is reversed when the angular frequency > 2, and the SDF is mainly characterized by elasticity in solution, nevertheless, the SDFM is predominantly elastic only at an angular frequency > 5. At small angular frequencies, the G′ and G″ are close to each other, while at large angular frequencies, G′ increases exponentially while G″ increases slowly in comparison. And the SDFM solutions exhibit lower elasticity compared to SDF solutions, and this difference in dynamic rheological properties between fibers is related to factors such as fiber structure, molecular chain size, and the monosaccharide composition.

Rheological test results show that SDFM has lower viscosity and elasticity, which makes SDFM more easily dispersed in the gut, helping to prolong the retention time of food in the gut [42]. And, lower viscosity and elasticity make SDFM more readily and fermented by bacteria, thus promoting the growth of beneficial bacteria such as *Akkermansia* and the production of SCFAs [75]. Meanwhile, low viscosity soluble dietary fibers usually have high water holding and swelling properties, which can absorb large amounts of water in the intestine, increase fecal volume, and promote intestinal peristalsis [76].

### 3.7. Effects of SDFs on DSS-Induced Anxiety and Depression-like Behavior in Mice

**Figure 5:**
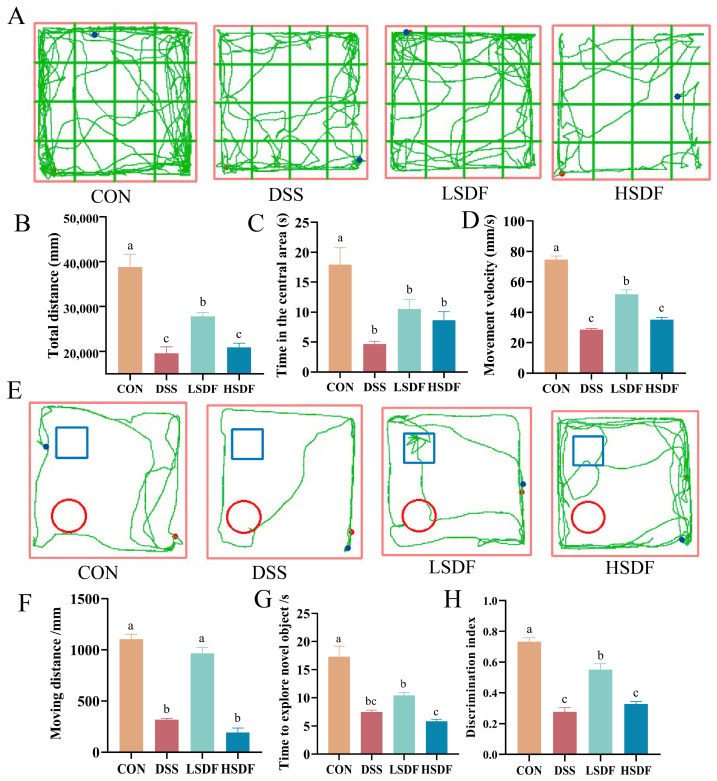
SDF intervention impacts cognitive behaviors following DSS-induced IBD mice. (**A**) Mouse trajectories in the open field test (OFT); (**B**–**D**) The moving distance, the time in the central area, and the movement velocity of the OFT test; (**E**) Mouse trajectories in new object recognition test (NORT); (**F**–**H**) The moving distance, time to explore novel object and discrimination index of the NORT test. Data are presented as mean ± SEM (*n* = 6). Statical analyses were carried out using one-way ANOVA along with Duncan’s multiple range test. Different letters indicate significant differences (*p* < 0.05).

Structural characterizations (scanning electron microscopy, Fourier transform infrared spectroscopy, particle size, X-ray diffraction, adsorption capacity, flow behavior) confirmed that cellulase-modified soluble dietary fiber (SDFM) exhibited better physicochemical properties than SDF: more porous microstructure; similar polysaccharide structure; more stable particle size distribution; higher crystallinity; better adsorption capacity; and lower viscosity. Therefore, we chose SDFM for further study, and examined the anxiolytic/antidepressant effects of LSDF (500 mg/kg) and HSDF (1000 mg/kg), we carried out several behavioral tests to assess the effects of SDFs. The specific trajectories of the mice in the OFT behavioral experiment are shown in Figure 5A. Then, the data were statistically analyzed. As shown in Figure 5B–D, compared to CON group, DSS treatment significantly reduced the total distance, time in the central area and movement velocity. However, compared with the DSS group, the total distance and movement velocity in the open field test (OFT) of LSDF group significantly increased (*p* < 0.05), while there was no significance between HSDF group and DSS group (*p* > 0.05). For a new object recognition test (NORT), the specific trajectories of the mice in the NORT behavioral experiment are shown in Figure 5E. And as shown in the Figure 5F–H, the moving distance and discrimination index of LSDF group were significantly higher than DSS group (*p* < 0.05), but there was no significance between the HSDF group and DSS group (*p* > 0.05).

**Figure 6:**
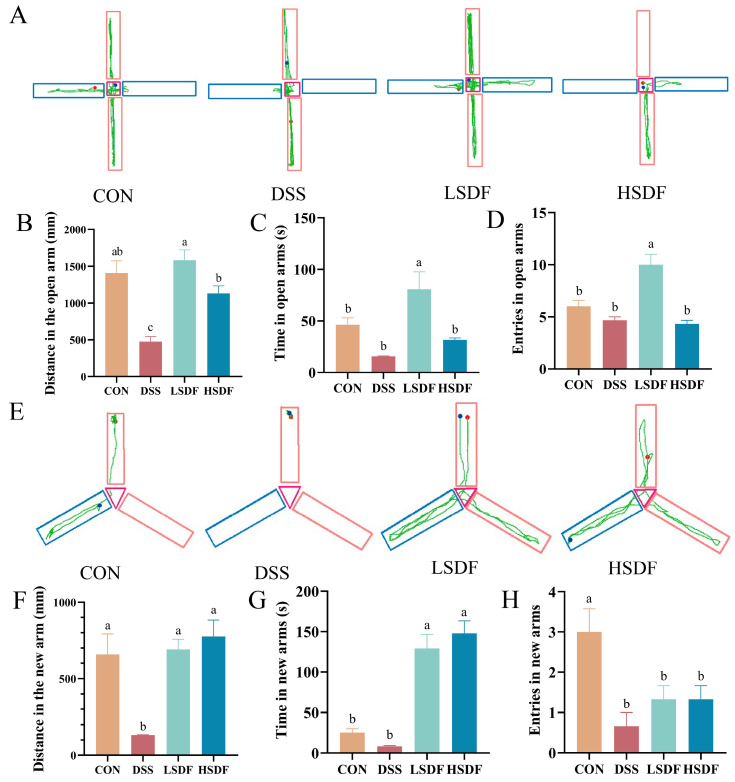
SDF intervention impacts cognitive behaviors following DSS-induced IBD mice. (**A**) Mouse trajectories in an elevated plus maze test (EPMT); (**B**–**D**) For the EPMT test, results including the distance, time, and entries in the open arms; (**E**) Mouse trajectories in the Y-Maze Test (YMT); (**F**–**H**) For the YMT test, results including distance, time, entries in the new arms. Data are presented as mean ± SEM (*n* = 6). Statical analyses were carried out using one-way ANOVA along with Duncan’s multiple range test. Different letters indicate significant differences (*p* < 0.05).

The specific trajectories of the mice in the elevated plus maze test (EPMT) and the Y-Maze Test (YMT) are shown in Figure 6A,E. In EPMT and YMT, the DSS group mice spent less time in the open arms, and made less distance and entries in the open arms or the new arms. However, the addition of LSDF significantly increased the time, distance and entries in the open arm (Figure 6B–D, *p* < 0.05), and the time and distance in the new arms of Y-Maze Test (YMT) were significantly increased in the LSDF and HSDF groups (Figure 6F,G, *p* < 0.05). And, there was generally no significant difference between the DSS group and the HSDF group in the time and entries in the open arms of EPMT and entries to the new arms in the YMT. Importantly, all these tests are widely used for screening the efficacy of repairing cognition in the DSS model of cognitive impairment [77]. According to the results above, it shows that LSDF is highly effective in DSS-induced cognitive impairment in mice, and 500 mg/kg SDF is more effective than 1000 mg/kg SDF at anti-depression.

### 3.8. Effects of SDFs on the Disease Parameters of DSS-Induced IBD Mice

**Figure 7:**
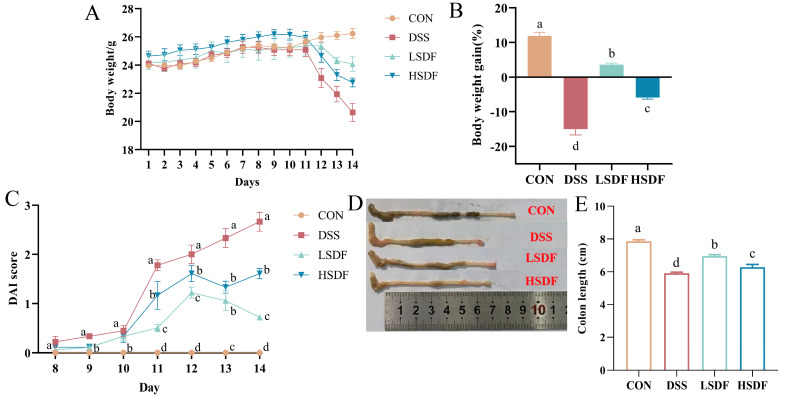
SDFs’ anti-inflammatory properties in vivo. (**A**) The change in body weights in different groups; (**B**) The body weight gain of different groups (the body weight of 14th day compared to 7th day); (**C**) Disease activity index (DAI) scores, significance analyses were performed for each group on the same day; (**D**) Images of mouse colons in different groups; (**E**) Colon length of different groups. Data are presented as mean ± SEM (*n* = 6). Statical analyses were carried out using one-way ANOVA along with Duncan’s multiple range test. Different letters indicate significant differences (*p* < 0.05).

When combined with the earlier structural characterization of SDFs, cellulase-treated SDFMs exhibit reduced viscosity and elasticity, increased crystallinity and adsorption, and other characteristics that allow SDFMs to exert their effectiveness in biological organisms more effectively than SDFs. Thus, we proceeded to examine how SDFM affected the mice’s IBD and cognitive decline brought on by DSS. In comparison to mice in the CON and SDFs groups, the body weight of DSS-induced IBD mice decreased considerably throughout the course of 7 days of DSS administration. On day 14, the body weight of mice with induced IBD decreased by 15.00 ± 2.96% compared to the end of acclimatization feeding on day 7. However, this impact was significantly reversed after the SDF intervention. The body weights of mice in the low-dose treatment group were more similar with the CON group compared to the high-dose SDF group (Figure 7A,B). DAI scores and colon length were utilized to evaluate the effectiveness of SDFs in protecting animals from DSS-generated colitis. DSS-induced inflammation in the colon of mice was often accompanied by a significantly shorter colon length compared to health mice [78]. After 7 days of DSS induction, the DAI scores of mice were noticeably greater than those of the CON group, with a maximum score of 2.67 ± 0.27 (Figure 7C). However, SDFs therapy was able to prevent the increase in DAI scores within 7 days. On day 14, DAI scores were significantly lower in the LSDF group (0.72 ± 0.12). According to the study’s findings, the DSS group’s colon length was noticeably less than that of the CON group (7.86 ± 0.20 vs. 5.92 ± 0.14 cm; *p* < 0.05), as shown in Figure 7D,E. After treatment with SDFs, the LSDF and HSDF group colon lengths were significantly longer compared with the DSS group (6.95 ± 0.19 vs. 6.28 ± 0.38 cm; *p* < 0.05), with the LSDF group of mice having significantly longer colon lengths.

**Figure 8:**
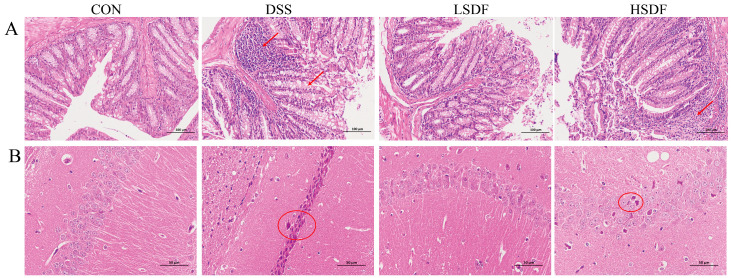
H&E-stained histopathological sections of colonic tissues, red arrow symbols indicate the severe deformation of the colonic villous structure, including the absence of cup cells and severe inflammatory cell infiltration phenomenon. Scale bars: 100 μm (**A**) and H&E-stained histopathological sections of brain tissues, red circles indicate neuronal degeneration and damaged nuclei or nuclear shrinkage. Scale bars: 50 μm (**B**).

The observations described previously showed that the therapeutic effects of LSDF was superior to HSDF. Furthermore, the present study was carried out using H&E staining to examine colonic and brain tissue sections for a wider examination to assess the effectiveness of LSDF and HSDF interventions (Figure 8A,B). As shown in Figure 8A, for the CON group, colonic sections indicated an intact structure with neatly arranged glands and crypts. However, there was a notable absence of cup cells, inflammatory cell infiltration, and severe mucosal ulcers in the colon sections of the DSS group. The SDF group showed a reduction in pathological changes caused by DSS, and in particular, the colonic structure of the LSDF group was more similar to the CON group than HSDF, indicating that LSDF had potent anti-inflammatory properties and was effective in reducing colitis in mice. Depression has been reported to be linked with inflammatory processes [79,80,81]. The histological alteration in the hippocampus was confirmed by the H&E staining of the brain. Figure 8B shows that nuclear shrinkage, nuclei damage and neuronal degeneration were increased in DSS-treated mice compared to the CON group. Supplementation with SDFs, particularly LSDF, had good relief for inflammation of the colon and brain.

### 3.9. Effect of SDFs on DSS-Induced Inflammation of the Gut and the Cerebral Cortex

**Figure 9:**
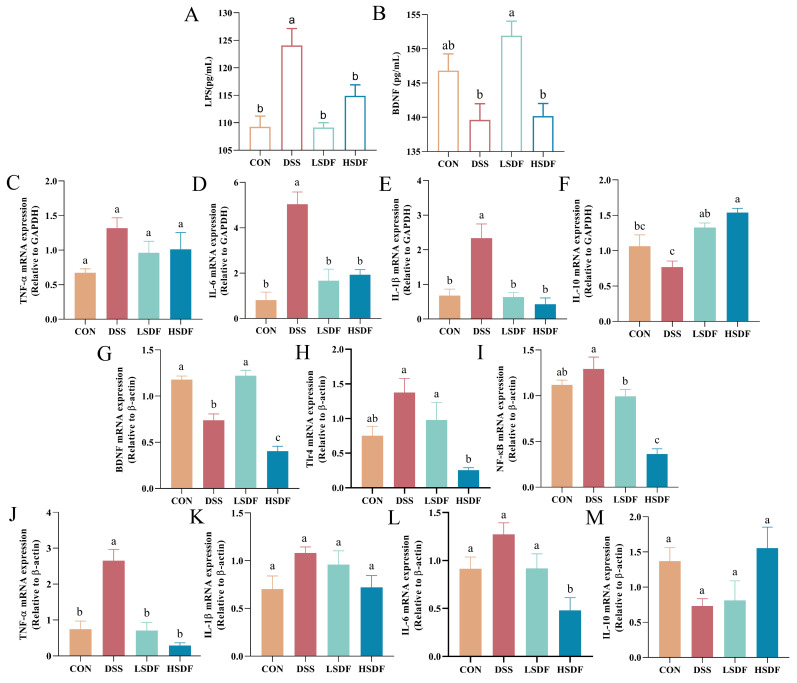
The inflammatory indicator level of the colon and brain: (**A**) Serum LPS; (**B**) Serum BDNF; (**C**–**F**) Relative mRNA expression of TNF-α, IL-6, IL-1β, and IL-10 in the colon; (**G**–**M**) Relative mRNA expression of BDNF, Tlr4, NF-κB, TNF-α, IL-1β, IL-6, and IL-10 in the brain. Data are presented as the mean ± SEM (*n* = 6). Statical analyses were carried out using one-way ANOVA along with Duncan’s multiple range test. Different letters indicate significant differences (*p* < 0.05).

The depression is commonly associated with abnormal changes in the level of inflammation. Our study found a significant reduction in serum levels of LPS after LSDF intervention (Figure 9A). Notably, LSDF treatment also significantly increased brain-derived neurotrophic factor (BNDF) levels of cerebral cortex (*p* < 0.05), while high doses of SDF showed almost identical levels of BDNF compared with the DSS group (Figure 9B,G). Consequently, we examined the pro-inflammatory cytokines to assess the degree of inflammation in the colon and brain tissues and found that mRNA levels of pro-inflammatory cytokines (TNF-α, IL-6, IL-1 β, Tlr4, and NF-kB) were significantly decreased in colon and brain tissues after the LSDF and HSDF administration (Figure 9C–E,H–L, *p* < 0.05), while significantly increasing the mRNA levels of IL-10 in colon (Figure 9F, *p* < 0.05) and brain (Figure 9M, *p* < 0.05). Overall, there was no significant difference between the LSDF and HSDF on colon inflammation modulation, but HSDF had a better reduction in inflammation in the brain.

### 3.10. Effect of LSDF on DSS-Induced Changes in Gut Microbiological Composition

In summary, for behavioral experiments (open field test (OFT), new object recognition test (NORT) and elevated plus maze test (EPMT)), LSDF showed a better alleviation of cognitive deficits than HSDF. The DSS-induced body weight of IBD mice showed significant weight loss, but the effect of weight loss after LSDF intervention was significantly reversed to a greater extent than that of HSDF. The LSDF group had a significantly lower DAI score than the HSDF group and had a longer colon length. Colon and brain tissue sections were assessed by H&E staining, LSDF group had a more similar colon and cerebral cortex structure to the CON group compared to the HSDF group. Biochemical analyses showed that LSDF was more effective than HSDF in decreasing the serum LPS levels and increasing the BDNF levels in the cerebral cortex, which may contribute to the improvement in colitis and cognitive impairment induced by DSS. Interestingly, HSDF performed better in reducing brain inflammation. Summarizing these previous findings, LSDF had overall better efficacy than HSDF.

**Figure 10:**
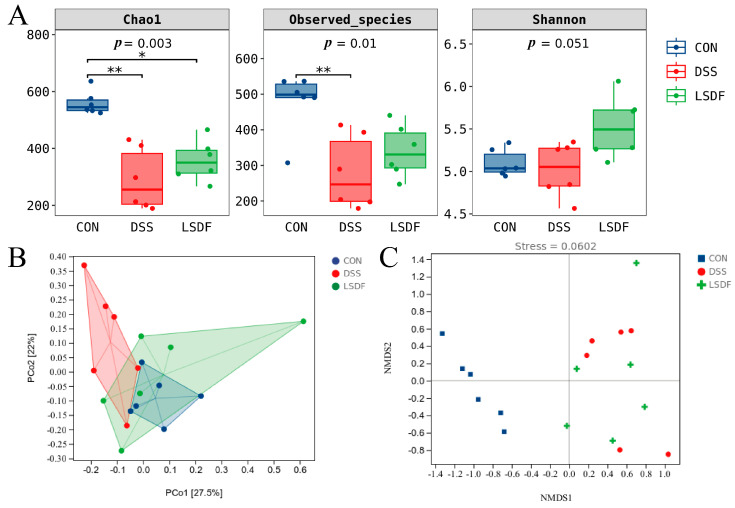
Effect of LSDF on the species diversity of the gut microbiota in DSS mice. (**A**) OTU level analysis in three groups, including chaos index, observed species, and Shannon index; *β*-diversity indices including PCOA (**B**) and NMDS (**C**). Data are presented as mean ± SEM (*n* = 6). Statical analyses were conducted using one-way ANOVA analysis, * *p* < 0.05, ** *p* < 0.01.

Gut microbiota plays an important role in maintaining gut barrier function, and its imbalance is recognized as a key factor in UC and cognitive impairment. We therefore further verified the alleviating effect of LSDF by investigating its modulating effect on gut microbiota. The 16S *r*RNA sequencing of cecum contents was conducted to validate the effect of LSDF intervention on the intestinal microbiota of mice with colitis. The α-diversity indices showed that the Chao 1 index and observed-species index of species richness in the DSS group mice significantly decreased compared to the CON group mice, while the trend was reversed after LSDF intervention (*p* < 0.05). Although the Shannon indices of LSDF mice showed certain increases compared to CON and DSS mice, there was no statistically significant difference. GM in the LSDF group was significantly improved by LSDF compared with those in the DSS group (Figure 10A).

*β*-diversity reflects differences in the species abundance and distribution between communities, and we analyzed *β*-diversity indices such as PCoA (Figure 10B) and NMDS (Figure 10C) between different groups. PCoA reveals the distinct clustering of the gut microbiota composition among the CON, DSS, and LSDF groups, which indicated that LSDF supplementation significantly changed the gut microbial populations. Thereafter, these changes among different groups were confirmed by the NMDS analysis. The significant intergroup variability between mice in the CON, DSS, and LSDF groups suggested the significant changes in the fecal microbiota of mice in the DSS-induced colitis disease state. The LSDF intervention deviated from the model group to a certain extent and converged to the CON group. These results indicate that, overall, there were significant differences in the gut microbial structure of mice in the CON and DSS groups, while the LSDF group converged toward the CON group.

**Figure 11:**
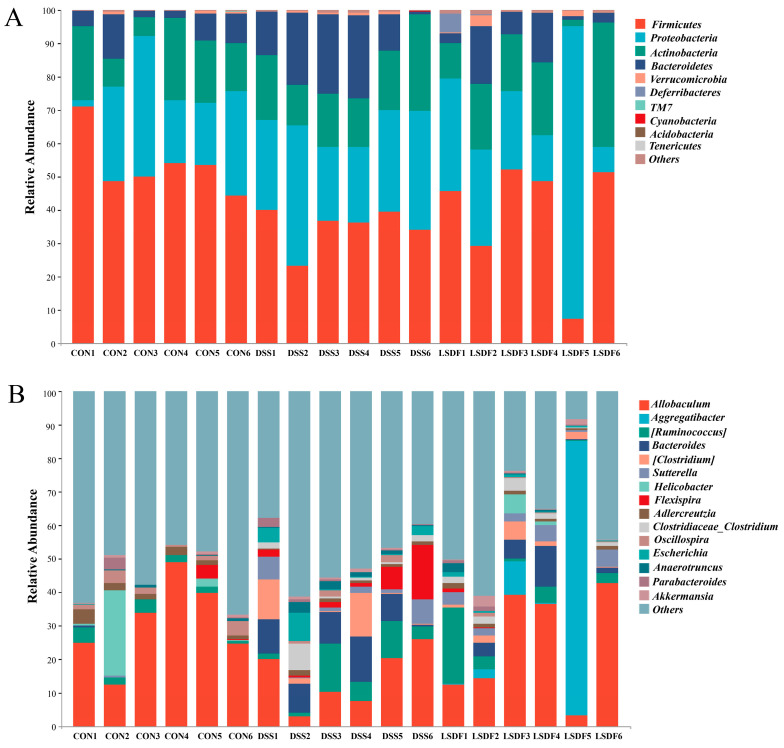
The effect of LSDF on the composition of the gut microbiota in mice. (**A**) The phylum-level relative abundance histogram of gut microbiota in three groups; (**B**) The genus-level relative abundance histogram of gut microbiota in three groups.

To evaluate the effect of LSDF intervention on the structure of intestinal microbiota, we compared the relative abundance of the gut bacteria in the CON, DSS, and LSDF groups of mice with colitis. At the phylum level, 10 primary phyla were defined in our study, DSS significantly increase the relative abundance of *Bacteroidetes* and decreased that of *Firmicutes*, *Verrucomicrobia*, and *Deferribacteres*, while the intervention of LSDF robustly increased the abundance of *Firmicutes*, *Proteobacteria*, *Verrucomicroia*, and *Deferribacteres* and decreased the ratio of *Bacteroidetes* (Figure 11A). At the genus level, the abundances of *Akkermansia*, *Allobaculum*, *Aggregatibacter*, *Helicobacter* in the cecum contents of mice were increased, while the abundances of *Bacteroides*, *Clostridium*, *Flexispira*, *Escherichia,* and *Anaerotruncus* were decreased after the LSDF intervention compared to the DSS group (Figure 11B). Overall, the DSS altered the composition of the intestinal flora and promoted the growth of harmful bacteria (*Flexispira*, *Bacteroides,* and *Escherichia*, etc.), whereas the LSDF intervention promoted the growth of beneficial bacteria (*Helicobacter* and *Aggregatibacter*, etc.).

**Figure 12:**
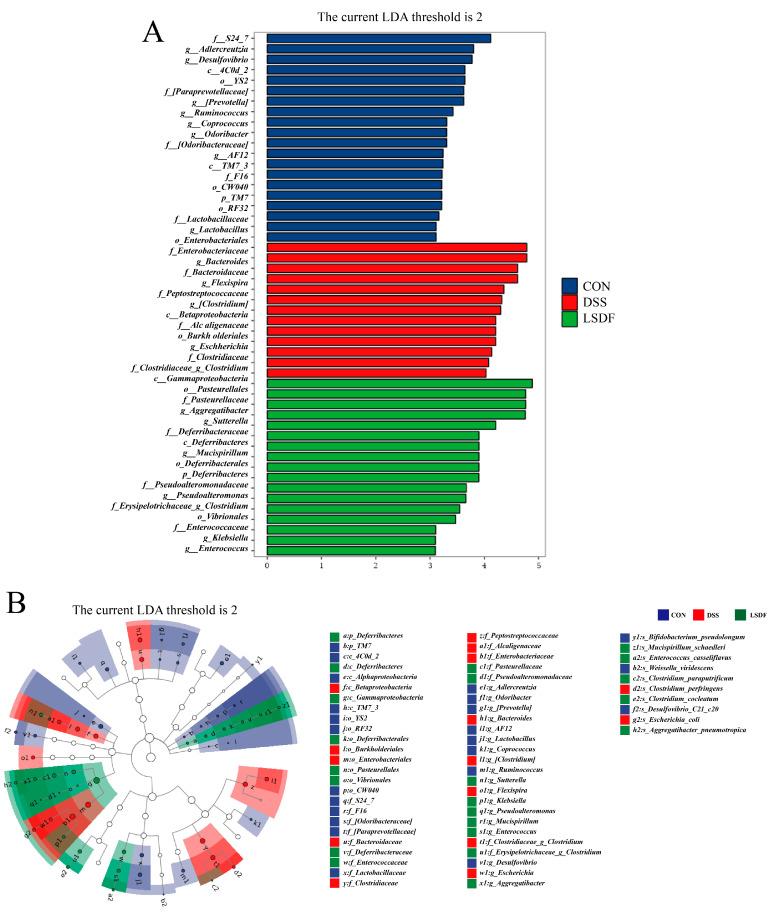
Effect of LSDF on the differentially abundant microbial composition of the gut microbiota in mice. (**A**) Scores for the abundances of different taxa using linear discriminant analysis (LDA). (**B**) Taxonomic cladogram obtained using LEfSe analysis.

Next, to define the characteristic bacteria of three groups, LEFSe analysis was performed from the phylum to genus levels with an LDA threshold of 2 (Figure 12A,B). The results indicated that a total of 50 OTUs were notably different among these three groups, among which there are 20, 13, and 17 significant differences in the CON, DSS, and LSDF groups, respectively. The mice in the CON group had 20 biomarkers, 3 of which were part of the phylum *Firmicutes*, 6 of which were part of the phylum *Bacteroidetes*, 4 of which were part of the phylum *Proteobacteria*, 4 of which were part of the phylum *TM7*, 2 of which were part of the phylum *Cyanobacteria,* and 1 of which were part of the phylum Actinobacteria. DSS mice had 13 biomarkers, 8 of which were part of the *Proteobacteria*, 3 of which were part of the phylum *Firmicutes,* and 2 of which were part of the phylum *Bacteroidetes*. LSDF mice had 17 biomarkers, 9 of which were part of the phylum *Proteobacteria*, 6 of which were part of the phylum *Deferribacteres,* and 2 of which were part of the *Firmicutes*. Particularly, *o_Enterobacteriales*, *g_Bacteroides,* and *g_Flexispira* were found to play an important role in the DSS treatment group; *c_Gammaprotebacteria*, *o_Pasteurellaceae,* and *g_Aggregatibacter* were found to play an important role in the supplementation of LSDF group under DSS condition. The results showed that there were significant differences in the community structure between the different groups, especially in the DSS group compared to the CON and LSDF groups, while the LSDF group was closer to the CON group, and the findings in Figure 12A are consistent with those in Figure 12B.

Overall, these findings imply that LSDF has a role in influencing gut microbial composition and altering its overall function.

### 3.11. Effect of SDFs on DSS-Induced Changes in Fecal SCFAs Levels

Research has indicated that an imbalance in the gut microbiota may result from colitis [82]. SCFAs are crucial for retaining the morphology of colonic epithelial cells, which is therefore utilized to effectively prevent intestinal diseases [83,84]. In this study, we used the gas chromatography to ascertain the short-chain fatty acid composition. The results indicated that the contents of SCFAs, including acetic acid, propionic acid, butyric acid, iso-butyric acid, valeric acid, and iso-valeric acid were low in the DSS group, and there was no obvious enhancement after LSDF treatment, except for iso-butyric acid (Figure S1). Furthermore, it was found that high dosages of soluble dietary fiber had a stronger intervention effect for acetic and propionic acids. In summary, the results showed that LSDF and HSDF promoted the production of SCFAs.

### 3.12. LSDF Altered the Metabolic Profile of DSS-Induced Mice

**Figure 13:**
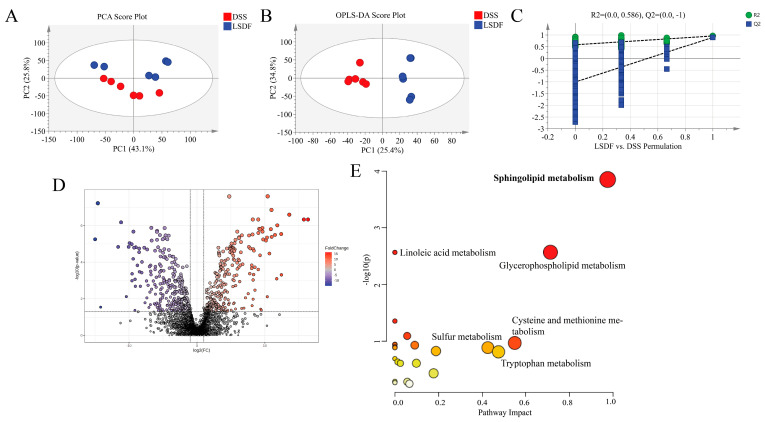
Effect of LSDF on brain metabolites in mice. (**A**) PCA based on mouse brain metabolites; (**B**) OPLS-DA based on mouse brain metabolites; (**C**) Diagram of a permutation test using the OPLS-DA method, where the number of tests was 200; (**D**) Metabolite volcano plots for the DSS and LSDF group; and (**E**) The metabolic pathways of brain differential metabolites between DSS and LSDF group.

To determine the mechanisms and pathways by which LSDF alleviates the symptoms of colitis-induced cognitive impairments, we conducted brain metabolomics experiments. We selected the LSDF group for metabolomics analysis and further discussion after comparing the intervention effects of LSDF and HSDF on the moderating impact of colitis and discovering that the effect of the LSDF was more substantial than HSDF. As a popular research approach in recent years, metabolomics can help us discover certain relevant biomarkers and gain a better understanding of the pathological processes and metabolic pathways of substances in the body. To explore whether LSDF ameliorates DSS-induced colitis through its metabolites, cerebral cortex tissues from the DSS and LSDF groups were collected for non-targeted metabolomics analysis. From the scores of multidimensional statistical analysis, including PCA (Figure 13A) and OPLS-DA (Figure 13B), the result showed that the loading plots of the LSDF group are clearly distinguishable from the DSS group. This indicates that, compared to the DSS group, the LSDF had a significant impact on brain metabolite composition. For the OPLS-DA test, when the number of permutation tests was 200, R2 = 0.586 and Q2 = −1 were obtained, which indicated that all OPLS-DA models were reliable without overfitting (Figure 13C). The results of the volcano map revealed significant alterations in brain metabolites after intervention with the LSDF, among which 294 were upregulated and 311 were downregulated (Figure 13D).

In this study, based on the OPLS-DA and *t*-test results, fold change ≥ 2 or fold change ≤ 0.05, VIP value > 1, and *p*-value < 0.05 were set as the differential metabolites screening conditions. To more intuitively demonstrate the pathways that the differential metabolites were enriched into between the DSS group and LSDF group, the KEGG pathways of the differential metabolites was mapped. The analysis of the KEGG pathway in the LSDF group vs. the DSS group revealed that the differentially abundant metabolites of the cerebral cortex identified herein were mainly involved in the sphingolipid metabolism, linoleic acid metabolism, glycerophospholipid metabolism, and so forth (Figure 13E).

**Figure 14:**
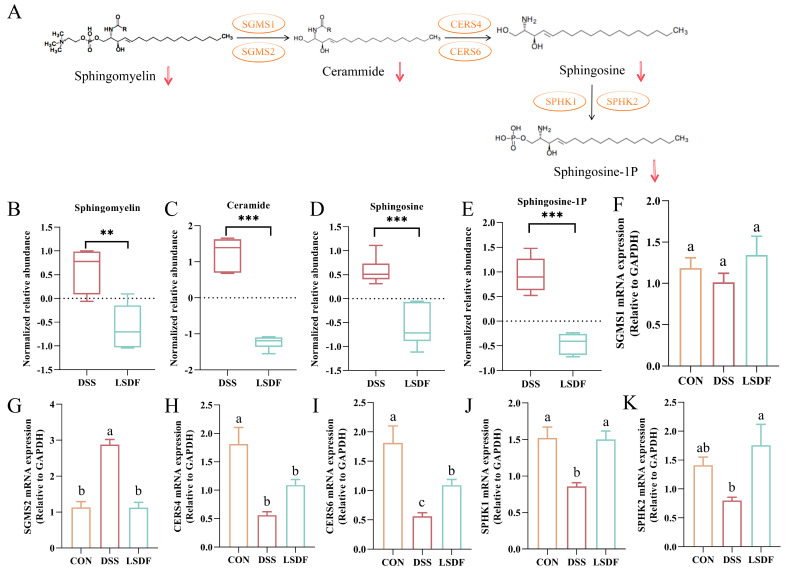
Impact of LSDF supplementation on the sphingolipid pathway of the brain in mice. (**A**) Mechanistic diagram of sphingolipid metabolic pathway (downward arrows indicate decreases in levels of relevant metabolites); (**B**–**E**) Normalized relative abundance of metabolites related to sphingolipid metabolic pathway: sphingomyelin (SM), ceramide (CER), sphingosine (SPH), and sphingosine-1P (S1P)—statistical analyses were carried out using two-tailed *t*-tests with Student’s *t*-tests; (**F**–**K**) mRNA expression of important genes in the sphingolipid metabolic pathway of brain: SGMS1, SGMS2, CERS4, CERS6, SPHK1, and SPHK2. Data are presented as mean ± SEM (*n* = 6). Different letters indicate significant differences (*p* < 0.05), ** *p* < 0.01, *** *p* < 0.001.

The mechanistic diagram of the sphingolipid metabolic pathway was shown in Figure 14A. In the mouse brain of LSDF group, significant declines were observed in the levels of certain small metabolites linked to sphingolipid metabolism pathways, such as sphingomyelin (SM), ceramide (SER), sphingosine (SPH), and sphingosine-1-P (S1P) compared to the DSS group (Figure 14B–E). Then, we used RT-qPCR to assess the expression levels of genes linked to the sphingolipid metabolism in order to validate the findings of metabolomics on the enrichment of metabolite pathways (Figure 14F–K). The intervention of LSDF may cause changes in the transcription factor levels associated with sphingolipid metabolism. Results indicated that the LSDF intervention significantly upregulated the expression of Sphingomyelin synthase 1 (SGMS1), Ceramide synthase 4 (CERS4), Ceramide synthase 6 (CERS6), Sphingosine kinase 1 (SPHK1), and Sphingosine kinase 2 (SPHK2) in the cerebral cortex, while significantly downregulating the expression of Sphingomyelin synthase 1 (SGMS2). This finding further demonstrated that the treatment with LSDF restored the imbalance in the sphingolipid metabolism caused by DSS.

### 3.13. Spearman Correlation Analysis Between Biochemical Indices, Behavioral Parameters, Brain Metabolites, and the Microbiota

**Figure 15:**
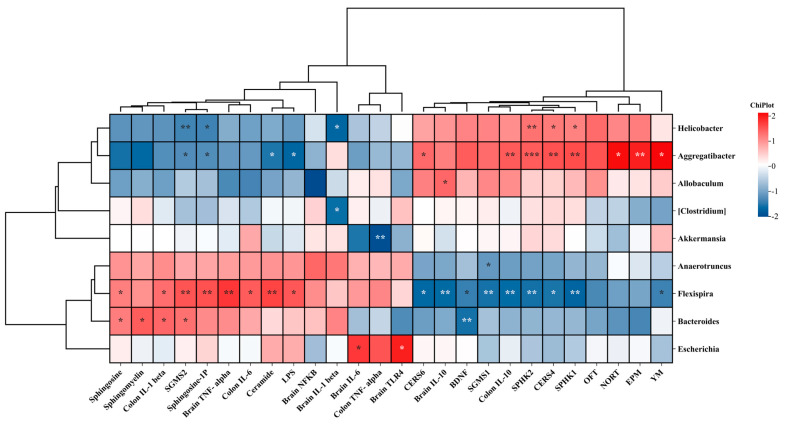
Spearman correlation between genus-level microflora and inflammatory indices of colon and brain, cognitive behavior, and the sphingolipid metabolism of brain. Statical analyses were conducted using *t*-test, * *p* < 0.05, ** *p* < 0.01, *** *p* < 0.001.

Based on the correlation of various parameters, Spearman correlation was used to validate the potential relationships between various parameters, including gut and brain inflammatory factors, behavioral experiments, brain sphingolipid metabolism and intestinal microbiota (Figure 15). In mice, *Flexispira*, *Bacteroides,* and *Escherichia* exhibited positive correlations with the levels of the sphingolipid metabolite (SM, ceramide, SPH, and S1P), the expression of mRNA SGMS2, inflammation factors of gut and brain (LPS, TLR4, IL-1β, TNF-α, IL-6, and TNF-α). On the other hand, it was negatively correlated with the expression of the sphingolipid pathway-related mRNA (SGMS1, CERS4, CERS6, SPHK1, and SPHK2), BDNF, anti-inflammatory IL-10, and cognitive behavior (OFT, NORT, EPM, YM). Interestingly, the *Helicobacter* and *Aggregatibacter* showed opposite results in contrast to the *Flexispira*, *Bacteroides,* and *Escherichia*.

## 4. Discussion

In the presented study, SDFs’ effects on DSS-induced colitis in mice and the accompanying behaviors of anxiety and depression have been assessed. It was found that the SDFs reduced the damage to the intestinal barrier. This effect’s mechanism may be related to the inhibition of inflammatory responses, which is in line with the results on inflammatory cytokines (TNF-α, IL-6, and IL-1β) in the brain and colon. Additionally, SDFs reversed some DSS-induced alterations in gut microbiota metabolites, including LPS and SCFAs. Moreover, SDFs remodeled the composition of the gut microbiome, and the abundance of beneficial bacteria was upregulated, and that of harmful bacteria was downregulated in DSS-induced IBD mice. At the same time, SDFs were found to improve synaptic plasticity in the brain by modulating the level of BDNF and sphingolipid-related metabolite, thereby preventing behavioral disorders, which suggests that gut–brain axis homeostasis may also be the underlying mechanism.

In recent years, the incidence of colitis has been increasing, accompanied by a greater probability that the patient will suffer from a psychiatric disorder, and it has been challenging to develop medications due to the intricate etiology. IBD and its accompanying anxiety and depression are among the gastrointestinal symptoms that are attributed to behavioral disorders by the gut–brain axis [85]. Evidence from both humans and rodents indicates the connection between intestinal barrier disruption and depression and anxiety. This damage may result in the release of some pathogens, including LPS, into the plasma, which may trigger neuroinflammation [86,87]. For instance, intestinal permeability is increased and serum LPS (the main external membrane constituent of Gram-negative bacteria) is increased by 3-fold in dementia patients with endotoxemia. According to the current studies, there is an increasing amount of evidence from sizable cohorts suggesting a connection between depressive or anxiety symptoms and the IBD clinical disease activity [88,89]. The prevalence of psychiatric disorders is also higher in patients with IBD: almost half of them experience anxiety symptoms, and one-third experience depressive symptoms [90]. More recent studies in rodents have shown that DSS-induced depressive-like and anxiety-like behaviors correlate with gut microbiota composition, apoptosis, synaptic damage, neuroinflammation, as well as BDNF levels [91,92,93]. As an important component of the gut-brain axis, gut microbiota plays an essential for cognitive function [94]. In conclusion, in behavioral experiments (including the open field test (OFT), the new object recognition test (NORT) and the elevated cross maze test (EPMT)), LSDF showed a better alleviation of cognitive deficits compared to the high dose (HSDF). And, biochemical analyses showed that LSDF was more effective than HSDF in decreasing the serum lipopolysaccharide (LPS) levels and increasing the brain-derived trophic factor (BDNF) levels in the cerebral cortex, which may contribute to the improvement in colitis and cognitive impairment induced by DSS.

As the fifth nutrient, dietary fiber has been increasingly found to provide important benefits to human health [95]. The structural characteristics, binding capacity, and nutrient transport capacity of dietary fiber are relevant to the functions it plays in the digestive tract [5]. More and more research in recent years has shown that dietary fiber can modulate cytokines [96,97], altering the structure of the gut microbiota [98] to alleviate intestinal inflammation, which has enormous potential for treating IBD [99]. It has also been reported that dietary fiber is more suitable for intervention in ulcerative colitis disease than Crohn’s disease [99,100]. A study revealed that celery SDF was more effective than IDF in alleviating colitis and reducing the interfering effects of flavonoids [101]. Millet soluble dietary fiber was found to alleviate DSS-induced colitis by increasing *lactobacilli* and F/B ratio to maintain intestinal microbiota balance [102]. In this study, the DSS-induced body weight loss was significantly lower in the IBD mice, but the recovery of body weight after the LSDF intervention was significantly better than that of HSDF. The LSDF group had a significantly lower disease activity index (DAI) than the HSDF group and a longer colon length. Colonic and brain tissue sections assessed by H&E staining showed that the LSDF group had colon structures that were more similar to those of the control group (CON), in contrast to the HSDF group. Taking these together, these findings suggest that LSDF is overall more effective than HSDF in terms of relieving symptoms associated with inflammatory conditions of the colon.

There is widespread agreement that the pathomechanism of ulcerative colitis is the interaction of exposure to the environment in genetically susceptible individuals, coupled with the dysbiosis of the intestinal microbiota, epithelial barrier defects, and immune dysregulation [11]. The intestinal epithelial barrier defects refer to the absence of cuprocytes, whereas cuprocytes secrete mucin-2 that provides a protective layer for the colon [103,104]. Recent studies indicated that intestinal permeability increases when the intestinal barrier is disrupted, while the metabolism and production of a number of pathogens, including LPS, are more likely to enter the bloodstream through the intestinal epithelium, which can exacerbate other inflammatory conditions, such as neuroinflammation in vivo [105,106,107]. The harm to the central nervous system (CNS) is linked to genetic susceptibility and environmentally induced shifts in the metabolite and protein levels [25,108]. Diets to improve cognitive impairment and prevent dementia are currently a major research hotspot today [109]. The cell wall component lipopolysaccharide (LPS) could induce exacerbated neuroinflammation by activating microglia, thereby inducing an increase in neuroinflammation, which is thought to be associated with cognitive decline [110,111]. In the present study, we found that SDFs significantly downregulated LPS levels in the serum of mice (*p* < 0.05), especially LSDF, which had a better effect compared to HSDF. Brain-derived neurotrophic factor (BDNF) has been found to be effective in enhancing the stress response triggered by Arg1 microglia in the hippocampus, thereby effectively alleviating depression [112]. In this study, we found that BDNF levels in the serum and brain tissues of mice with colitis were significantly increased after LSDF intervention (*p* < 0.05), while there was no significant difference in the BDNF levels after the HSDF intervention compared with the DSS group (*p* > 0.05), which suggests that the LSDF intervention is more effective in increasing BDNF levels and thus alleviating depression.

Cytokines refer to a series of small heterogeneous peptides which can affect cell proliferation and differentiation as well as inflammation, as evidenced by studies that the study of these cytokines is important for psychiatric disorders like depression and anxiety [113], of which IL-1β has been implicated in the etiology of depressive-like behaviors, and in conjunction with IL-6 and TNF, affects depressive disorders [114]. Some studies have shown that IL-1β levels in depressed patients are elevated and positively correlated with the degree of illness in elderly depressed patients and in women with postpartum depression [115,116]. Interleukin-6 is a neuronal growth factor that has been linked to depressive symptoms such as low mood and decreased appetite [117], and several studies have demonstrated elevated levels of IL-6 in depressed patients [118,119]. TNF-α levels have been found to be positively correlated with major depressive disorder (MDD) severity in serum and have a predictive value [120]. Studies have shown that adult C57BL/6 mice fed high dose of pectin have decreased levels of TNF-α, IL-1β, and IL-6 in their hippocampus while brain-derived neurotrophic factor levels are increased, thereby reducing neuroinflammation and affecting mood- and cognition-related brain regions [121]. TLR4 is a pattern recognition receptor that plays an important role in the innate immune system, recognizing pathogen-associated molecular patterns (PAMPs), such as bacterial LPS [122]. The activation of TLR4 can set off signaling pathways that lead to the development of pro-inflammatory factors (TNF-α, IL-1β, and IL-6) that trigger inflammation [123]. NF-κB is a protein complex involved in the expression of various genes encoding inflammatory factors and immune response regulators, and its activation has been associated with a variety of inflammatory diseases [124]. It has been proved that treatment with SCFAs can reduce intestinal inflammation by decreasing NF-κB signaling pathway and upregulating the expression of the anti-inflammatory cytokine IL-10 [125]. Over all, there was no significant difference in serum inflammation levels (TNF-α, IL-6, IL-1β and IL-10) between the LSDF and HSDF groups. Interestingly, HSDF performed better in reducing brain inflammation (Tlr4, NF-κB, IL-6, and IL-10).

There is growing evidence that imbalances in the gut microbiota’s structure are strongly linked with increased intestinal permeability, induced intestinal inflammation, and peripheral blood inflammation, increased blood–brain barrier permeability, and increased central inflammation, which leads to neurological dysfunction [126,127]. Studies have confirmed that the restoration of gut microbiota structure can increase intestinal permeability and ameliorate abnormal cognitive behavior in mice [23]. At the phylum level, it has been investigated that *Firmicutes* can preserve the integrity of the intestinal barrier by upregulating the tight junction protein and activating the Akt/MTOR signaling pathway, playing an essential role in the mouse models of UC [128]. In addition, studies have shown that *Firmicutes* improved memory scores in Alzheimer’s disease models and human subjects with self-reported memory problems, and in a trial of healthy adults with MCI, *Firmicutes* also significantly improved cognitive function [129]. The changes in gut microbes have been linked to the development of UC. Specifically, there is research which has found that the elevated abundance of Bacteroides in IBD patients may be related to the pathogenesis of colitis [130], which aligned with our study results. Additional analysis was performed on the species composition at the genus level. After DSS intervention, there were large changes in microbiota abundance, and LSDF reversed these changes, including the abundance of harmful bacteria like *Clostridium*, *Flexispira*, *Escherichia,* and *Anaerotruncus* decreased, while the abundance of *Allobaculum*, *Aggregatibacter*, *Helicobacter* and *Akkermansia* and other beneficial bacteria increased. It was found that *Escherichia* increased the level of intestinal inflammation by secreting TNF-α and IL-6 [131]. Studies revealed that *Akkermansia* improves cognitive dysfunction by regulating BDNF and inflammation levels in the gut-brain axis [132,133]. The currently recognized possible mechanisms of *Akkermansia* in the treatment of colitis are the increased production of SCFA, promoted pro-inflammatory cytokines, and the altered composition of the intestinal microbiota [134]. In addition, another study found that silibinin increased the level of *Allobaculum* and *Akkermansia*, and then alleviated memory deficits in AD rats, and decreased amyloid plaque deposits in the brain, which was consistent with the results of our study [135].

Previous research has proven that SCFAs can modulate intestinal permeability, inhibit the release of pro-inflammatory molecules, and ultimately alleviate ulcerative colitis [136]. Our findings indicated that the six types of SCFAs—acetic acid, propionic acid, butyric acid, iso-butyric acid, valeric acid, and iso-valeric acid—that are metabolites of dietary fiber digested by the microbiota were significantly elevated under the SDFs intervention. Research has demonstrated that SDF typically exerts beneficial effects through the regulation of systemic energy homeostasis by SCFAs [137]. Recently, results have shown that the gut microbiome can generate SCFAs through the phylum *Firmicute*, *Actinobacteria,* and *Bacteroidetes*. Some studies have found that UC patients have a lower abundance of the butyrate-producing genera *Faecalibacterium prausnitzii* and *Roseburia hominis* from the phylum *Firmicutes* [138,139]. The growth of these beneficial bacteria and the production of short-chain fatty acids (SCFAs) may be influenced by the structural features of SDFM: surfaces with more cracks and pores may provide more attachment sites and protection for bacteria, thus promoting the growth of beneficial bacteria; hydrolysis by cellulase to produce oligosaccharides or monosaccharides, which can act as fermentation substrates for gut bacteria; the higher crystallinity of SDFM affects its solubility and fermentability in the gut; the lower viscosity and elasticity of SDFM may be more accessible and fermentable by bacteria, thus promoting the growth of beneficial bacteria and the production of SCFAs.

Furthermore, in this study, the KEGG pathway analysis of brain metabolites in mice also revealed that the sphingolipid metabolism was the metabolic pathway with the largest pathway impact factor after LSDF intervention. It has been found that Saikosaponin enhances the sphingolipid metabolism in the cerebral cortex through apolipoprotein E, which leads to neurovascular coupling and exerting its antidepressant effects [140]. Metabolomics analysis in this study revealed that DSS-induced cognitive deficits in mice as well as intestinal microbiota dysbiosis, which also resulted in dysregulated sphingolipid metabolism. Consistent with our results, several clinical trials have demonstrated that the dysregulation of the sphingolipid metabolism is a significant aspect of dysfunction in patients with depression [141,142]. Notably, under DSS, mice brain showed lipid metabolism disorders (the level of SM, CER, SPH, and S1P in the brain of mice increases) which implies that the pathophysiology of cognitive impairment involves DSS-mediated sphingolipid metabolism. After the intervention of LSDF, the abnormal sphingolipid metabolism can be alleviated by regulating the expression level of genes related to sphingolipid metabolic pathway (SGMS1, SGMS2, CERS4, CERS6, SPHK1, and SPHK2), thus alleviating the dysfunction of the central nervous system of mice and alleviating the cognitive and emotional abnormalities. Sphingolipid is a kind of amphoteric lipid containing sphingosine skeleton, which is an important structural component of cell membrane containing SM, CER, SPH, and other substances, which participates in various cellular processes, such as cell interaction, cell proliferation, migration, differentiation, and apoptosis [143]. Sphingolipid metabolism has been reported to participate in some pathophysiological mechanisms of depression, including inflammation, neurodegeneration, and HPA axis activation [144]. The level of SM is crucial for cell function, which is the fundamental component of the plasma membrane [145], and SM has been proposed as a novel target for antidepressant treatment [146]. In addition to forming the structure of cell membrane, CER also plays a major role in the physiological processes of differentiation and cell growth [147]. The sphingomyelin (SM)-ceramide (CER) pathway has been found to be an important regulator of neurodegenerative diseases [148,149]. TNF-α induces the formation of ceramides, which mediate apoptosis. Ceramide metabolites (Sphingosine-1-P (S1P)) play an important role in inflammation by inhibiting ceramide-mediated apoptosis through the activation of extracellular pathways, such as extracellular signal-regulated kinases (ERKs) [150,151]. In addition, sphingosine (SPH), a by-product of ceramide commonly associated with cellular stress response and apoptosis, can be further phosphorylated by sphingosine kinase to produce S1P [152]. The results of this study have important implications for understanding the antidepressant mechanism of LSDF, which may be linked to regulating the sphingolipid metabolism to alleviate the symptoms of DSS mice.

In this study, we found that the intervention of SDFs from lentil hulls significantly alleviated anxiety-like and depression-like behaviors in mice with DSS-induced colitis. Moreover, SDFs exerted a prominent effect on reducing intestinal barrier damage, serum LPS levels, and inflammation in both the gut and brain. We also noted that the levels of BDNF were enhanced in mice supplemented with LSDF. Notably, LSDF demonstrated superior intervention effects compared to HSDF. The further analysis of the gut microbiota showed a decrease in the abundance of harmful bacteria such as *Bacteroides*, *Clostridium*, *Flexispira*, *Escherichia*, and *Anaerotruncus*, and an increase in the abundance of benefit bacteria such as *Allobaculum, Aggregatibacter*, *Helicobacter,* and *Akkermansia* after LSDF intervention. Moreover, the metabolomics results showed that LSDF affected the metabolic profile of brain tissue mainly through the sphingolipid metabolic pathway, and the preliminary validation of the sphingolipid pathway was obtained by liquid–liquid chromatography and RT-PCR. Furthermore, Spearman correlation analysis indicated that harmful bacteria (*Flexispira*, *Bacteroides,* and *Escherichia*) were positively correlated with gut and brain inflammatory factors and metabolites related to the sphingolipid pathway, while the expression levels of genes related to the sphingolipid pathway, the brain-derived trophic factor BDNF, the anti-inflammatory factor IL-10, and cognitive behaviors (OFT, NORT, EPM, YM) became negatively correlated, while the opposite results were observed for beneficial bacteria (*Helicobacter* and *Aggregatibacter*), supporting the beneficial role of LSDF in mitigating DSS-induced colitis, anxiety, and depression. Overall, our study demonstrated that LSDF exerts anti-inflammatory and antidepressant effects in DSS-induced IBD mice by modifying the structural composition of the intestinal bacteria and brain sphingolipid metabolism. However, there were differences between the DSS model and human IBD [153,154], although DSS-induced colitis is the most popular and flexible model for preclinical IBD studies, it is not an exact replica of human colitis and some of the results obtained from this model cannot be directly applied to humans [155]. In addition, the association of DSS with SCFAs in the lumen of the colon or sphingolipid metabolites in the brain may be quite different from the gut–brain axis of humans. Further subsequent studies in human subjects are needed to confirm these effects.

## 5. Conclusions

This study evaluated the effects of lentil hull soluble dietary fibers (SDFs) on DSS-induced colitis and associated anxiety and depression in mice. Structural characterizations revealed that cellulase-modified soluble dietary fiber (SDFM) had a more porous microstructure, a more stable particle size distribution, higher crystallinity, better adsorption capacity, and lower viscosity compared to soluble dietary fiber (SDF). Then, we chose SDFM for animal experiments. The results showed that SDFM significantly reduced the anxiety-like behaviors, intestinal barrier damage, and inflammation in both the gut and brain. LSDF outperformed HSDF in improving cognitive deficits, reversing weight loss, lowering DAI scores, and restoring colon length and tissue structure, with better effects on serum LPS and BDNF levels. Further study showed that LSDF modulated gut microbiota by decreasing harmful bacteria (e.g., *Bacteroides*, *Escherichia*) and increasing beneficial species (e.g., *Akkermansia*, *Helicobacter*). Metabolomics analysis revealed that LSDF altered brain metabolism primarily via the sphingolipid pathway. At last, the Spearman correlation analysis indicated that beneficial bacteria were negatively correlated with inflammatory markers and sphingolipid metabolites, while harmful bacteria showed positive correlations. These findings provide novel insights into the mechanism by which soluble dietary fiber maintains the balance of the gut–brain axis and highlights the potential benefit of lentil hull soluble dietary fiber in both the prevention and therapy of colitis and its neuropsychiatric complications. Future studies can consider the inclusion of female mice to better understand potential sex-specific mechanisms underlying the relationship between colitis and cognitive function. Also, future clinical trials are needed to confirm the efficacy and safety of SDF in humans with colitis and cognitive impairment, providing the basis for investigating the therapeutic potential of SDF in human IBD.

## Data Availability

The original contributions presented in the study are included in the article, further inquiries can be directed to the corresponding author.

## Supplementary Materials

The following supporting information can be downloaded at: https://www.mdpi.com/article/10.3390/foods14050870/s1, Table S1: Disease activity index (DAI) scoring criteria; Table S2: Primer sequence for quantitative PCR; Table S3: The gradient elution of the Q Exactive Focus system; Figure S1: The levels of SCFA metabolites in contents of the cecum.
