# Supplementary material for: Supplementation with Lentil (Lens culinaris) Hull Soluble Dietary Fiber Ameliorates Sodium Dextran Sulfate-Induced Colitis and Behavioral Deficits via the Gut-Brain Axis
Source: Foods. 2025 Mar 3;14(5):870. doi: 10.3390/foods14050870 (PMC11898428; doi:10.3390/foods14050870)
Supplement: Supplementary file 1 [file foods-14-00870-s001.zip › foods-3493039-supplementary.pdf]

Supplementary Materials

Table S1. Disease activity index (DAI) scoring criteria.

Score	Rectal bleeding	Stool consistency	Weight loss
0	normal	normal	<1%
1	occult blood	loose but formed stools	1-5%
2	slight blood	loose stools	6-10%
3	bleeding	slight diarrhoea	11-15%
4	abundant bleeding	watery diarrhoea	> 15%

Table S2. Primer sequence for quantitative PCR.

Primer	Forward primer	Reverse primer
BDNF	5'-TCATACTTCGGTTGCATGAAGG-3'	5'-ACACCTGGGTAGGCCAAGTT-3'
Tlr4	5'-ATGGCATGGCTTACACCACC-3'	5'-GAGGCCAATTTTGTCTCCACA-3'
IL-6	5'-TCTATACCACTTCACAAGTCGGA-3'	5'-GAATTGCCATTGCACAACTCTTT-3'
IL-10	5'-CTTACTGACTGGCATGAGGATCA-3'	5'-GCAGCTCTAGGAGCATGTGG-3'
NF-κB	5'-CATCACCCTGGGCTTCTTCCT-3'	5'-TGGGCTCCAATCCTGTCAATC-3'
TNF-α	5'-CCCTCACACTCAGATCATCTTCT-3'	5'-GCTACGACGTGGGCTACAG-3'
IL-1β	5'-GCAACTGTTCCTGAACTCAACT-3'	5'-ATCTTTTGGGGTCCGTCAACT-3'
SGMS1	5'-GAAGGAAGTGGTTTACTGGTCAC-3'	5'-GACTCGGTACAGTGGGGGT-3'
SGMS2	5'-GAGACAGCAAAACTTGAAGGTCA-3'	5'-CCCGTTGGATAAGGTCTTGGG-3'
CERS4	5'-TACCCACATCAGACCCTGAAT-3'	5'-TGAAGTCCTTGCGTTTGACATC-3'
CERS6	5'-GATTCATAGCCAAACCATGTGCC-3'	5'-AATGCTCCGAACATCCCAGTC-3'
SPHK1	5'-ATGGAACCAGTAGAATGCCCT-3'	5'-TCCGTTCGGTGAGTATCAGTTTA-3'
SPHK2	5'-CACGGCGAGTTTGGTTCCTA-3'	5'-CTTCTGGCTTTGGGCGTAGT-3'
GAPDH	5'-AGACCCACACTTCTCCATTTCC-3'	5'-TGAAATGTGCACGCACCAAG-3'

Table S3. The gradient elution of the Q Exactive Focus system.

Time(min)	Flow velocity(mL/min)	A(0.1% formic acid water)	B(acetonitrile)
0	0.4	90	10
5	0.4	70	30
10	0.4	20	80
12	0.4	15	85
15	0.4	5	95
18	0.4	5	95
20	0.4	90	10
25	0.4	90	10

Acetonitrile (B) and formic acid (A) at 0.1% made up the mobile phase. The mobile phase flow rate was 0.4 mL/min, the injection volume was 2 μL. The specific gradient elution settings: 0-5min, 90%-70% A, 10%-30%B; 5-10min, 70%-20% A, 30%-80%B; 10-12min, 20%-15% A, 80%-85%B; 12-15min, 15%-5% A, 85%-95%B;15-18min, 5%-5% A, 95%-95%B;18-20min, 5%-90% A, 95%-10%B;20-25min, 90%-90% A, 10%-10%B.

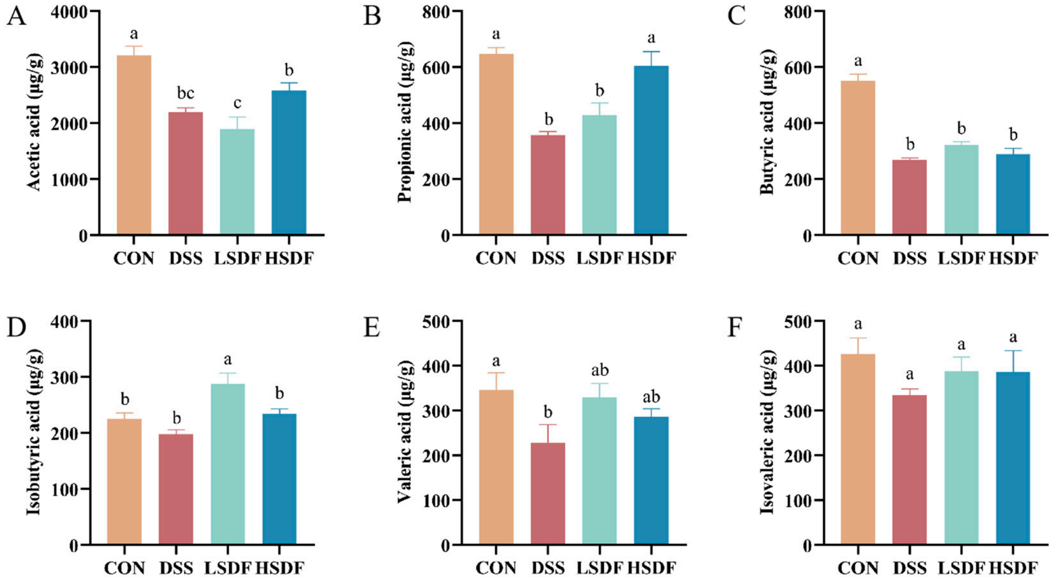

Figure S1. The levels of SCFA metabolites in contents of the cecum. (A)Acetic acid; (B)Propionic acid; (C)Butyric acid; (D)Isobutyric acid; (E)Valeric acid; (F)Isovaleric acid. Data are presented as mean ± SEM (*n* = 6). Statical analyses were carried out using one-way ANOVA with Duncan's multiple range test. Significant differences are indicated by different letters ($p < 0.05$).
